# Innate Immune Sensing of Influenza A Virus

**DOI:** 10.3390/v12070755

**Published:** 2020-07-14

**Authors:** Gaurav Malik, Yan Zhou

**Affiliations:** 1 Vaccine and Infectious Disease Organization-International Vaccine Centre (VIDO-InterVac), University of Saskatchewan, Saskatoon, SK S7N 5E3, Canada; gam123@mail.usask.ca; 2 Department of Veterinary Microbiology, Western College of Veterinary Medicine, University of Saskatchewan, Saskatoon, SK S7N 5B4, Canada

**Keywords:** influenza virus, innate immune response, pattern recognition receptors

## Abstract

Influenza virus infection triggers host innate immune response by stimulating various pattern recognition receptors (PRRs). Activation of these PRRs leads to the activation of a plethora of signaling pathways, resulting in the production of interferon (IFN) and proinflammatory cytokines, followed by the expression of interferon-stimulated genes (ISGs), the recruitment of innate immune cells, or the activation of programmed cell death. All these antiviral approaches collectively restrict viral replication inside the host. However, influenza virus also engages in multiple mechanisms to subvert the innate immune responses. In this review, we discuss the role of PRRs such as Toll-like receptors (TLRs), Retinoic acid-inducible gene I (RIG-I), NOD-, LRR-, pyrin domain-containing protein 3 (NLRP3), and Z-DNA binding protein 1 (ZBP1) in sensing and restricting influenza viral infection. Further, we also discuss the mechanisms influenza virus utilizes, especially the role of viral non-structure proteins NS1, PB1-F2, and PA-X, to evade the host innate immune responses.

## 1. Introduction

The constant challenge to living cells by invading pathogens drives the evolution of innate immune systems to rapidly detect and respond to non-self molecules, such as virus-derived nucleic acids [1]. Essential to the host’s innate immune responses to pathogens is to differentiate non-self molecules from self molecules, which is executed through pattern recognition receptors (PRRs) that recognize pathogen-associated molecular patterns (PAMPs), and in some cases, danger-associated molecular patterns (DAMPs) released by the infected host [2].

Influenza A virus (IAV) infects a wide variety of species and is an important human respiratory pathogen that causes annual epidemics and occasionally pandemics, posing severe public health concerns. The major difficulty in defending against IAV infection is the high genetic variability of the virus allowing the rapid generation of antigenically drifted and shifted, as well as reassortant viruses that can escape the acquired immunity against previous virus strains, or gain resistance to antiviral agents. The constant changing nature of IAVs poses challenges on vaccine development. While the antigenic drift requies annual vaccine updates to match the vaccine candidate virus to the predicted circulating virus, the antigenic shift results in the emergence of novel viruses, which requires new vaccine development. Thus, the innate immune response to IAV, which acts in the absence of adaptive immunity, plays a pivotal role in controlling IAV infection. In this review, we highlight the mechanisms that govern influenza virus recognition by various PRRs, the effects that follow such recognition, and the strategies employed by the virus to evade the innate immune recognition.

## 2. Influenza A Virus (IAV)

IAV belongs to the family Orthomyxoviridae; its genome contains eight segmented negative-strand RNA molecules encoding for at least 20 proteins [3]. Each segment exists in the form of vRNP, consisting of the viral RNA (vRNA) molecule associated with viral polymerase proteins (polymerase basic (PB) 1 and 2, and polymerase acidic (PA)) and nucleoprotein (NP) [4]. The IAV life cycle is centered on vRNP, which is responsible for viral replication (from viral RNA (vRNA) to complementary RNA (cRNA), and from cRNA to progeny vRNA) and transcription (from vRNA to mRNA). The IAV enters the respiratory epithelial cells by binding the sialylated glycoconjugates on host cell receptors via viral hemagglutinin (HA) which triggers virion endocytosis. The endocytosis occurs either in a clathrin-dependent manner or via micropinocytosis [5,6]. The low pH in endosome then activates the viral M2 ion channel while also triggering conformational changes in viral HA, leading to the exposure of the fusion peptide in the HA2 region for its insertion into the endosomal membrane. The activation/opening of the M2 ion channel acidifies the viral core releasing the packaged vRNPs from viral M1 that makes their way into the cytoplasm following HA mediated fusion of viral and endosomal membranes [7,8]. The exposed vRNPs possess nuclear localization signals (NLS), and are thus recognized by adaptor protein importin-α which recruits importin-β, resulting in vRNPs import to host nucleus [9,10,11,12,13,14]. Once inside the nucleus, viral RNA replication and transcription are ensured by viral RNA-dependent RNA polymerase. The viral M1 and nuclear export protein (NEP) then aid in the export of synthesized vRNPs to the cytoplasm via the CRM1 (Chromosomal Maintenance 1, also known as Exportin 1) nuclear export pathway. In this process, the viral NEP crosslinked to vRNPs via M1, interacts with CRM1, thus shuttling vRNPs from nucleus to cytoplasm [15,16,17]. These vRNPs are finally assembled into virions that bud from the infected cells and then spread to other non-immune as well as immune cells such as macrophages in the respiratory tract. The replication cycle of the influenza virus is very quick, with the first viral shedding from infected cells observed as early as 6 h [18,19].

Influenza virus infection is recognized by a variety of PRRs: the Toll-like receptors, Retinoic acid-inducible gene I (RIG-I), the NOD-like receptor family member NOD-, Leucine-rich repeat (LRR)-, pyrin domain-containing 3 (NLRP3), and the Z-DNA binding protein 1 (ZBP1). These PRRs provide viral recognition in distinct cellular compartments of different cell types and at different stages of infection. The activation of these PRRs results in mainly two arms of host defense mechanisms, IFN-mediated antivial response and IL-1β-mediated inflammation, which present a coordinated front in warfare against influenza virus infection.

## 3. Toll-Like Receptors (TLRs)

**Figure 1:**
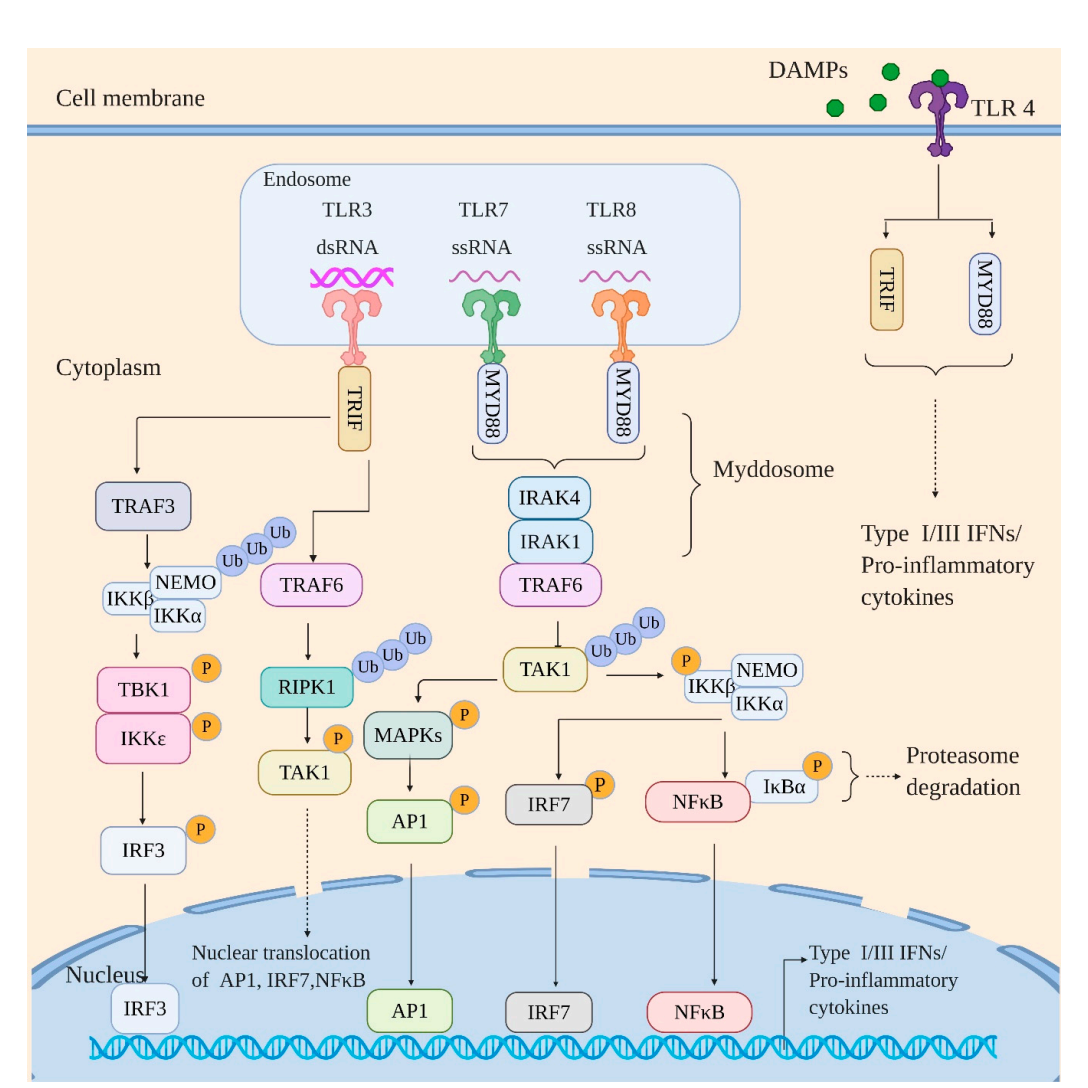
Toll-like receptor (TLR) signaling in response to viral infection. The TLRs engage the viral RNA signal in a MyD88-dependent (TLR7 and TLR8) and TRIF-dependent manner (TLR3). The MyD88 dependent pathway proceeds via formation of “Mydossome” with TRAF6 activating TAK1 kinase via polyubiquitination. The activated TAK1 activates IKK kinase complex and various MAP kinases by phosphorylation. The activation of IKK complex leads to the activation and nuclear translocation of NF-κB (by targeting inhibitor IκBα for proteasomal degradation) and IRF7; the MAPK kinases, however, activate AP-1 family of transcription factors followed by their nuclear translocation. The TRIF-dependent pathway, on the other hand, directly recruits TRAF3 and TRAF6. The TRAF6 then activates RIPK1 by polyubiquitination, which in turn activates TAK1 by phosphorylation leading to activation and nuclear translocation of NF-κB, IRF7, and AP-1 family of transcription factors. TRAF3 on the other hand activates IKK complex by polyubiquitination which in turn activates IKKε/TBK1 by phosphorylation leading to activation and nuclear translocation of IRF3. Of note, TLR4 recognizes endogenous danger-associated molecular patterns (DAMPs) secreted by influenza-infected cells and signal via both adaptors, MyD88 and TRIF. Signaling by various TLRs thus culminates with the induction of interferons and proinflammatory cytokines.

Mammalian TLRs represent the first identified class of PRRs and obtain their name from the product of the Toll gene in Drosophila [20]. The human TLR family consists of 10 members (TLR 1-10) among which TLR 3, 7, and 8 recognize viral RNA. TLRs are primarily confined to the cell surface and/or endosome and share a common architecture: an N-terminal leucine-rich repeat (LRR)-containing domain, a transmembrane domain, and a C-terminal cytoplasmic Toll/IL-1 receptor (TIR) domain [21]. The LRR-containing domain is responsible for ligand binding, whereas the cytoplasmic TIR domain recruits downstream signaling adaptors. As shown in Figure 1, all the TLRs generally signal via an adaptor protein, myeloid differentiation primary response protein 88 (MyD88) with an exception to TLR3 which exclusively requires TIR-domain-containing adapter-inducing interferon-β (TRIF) adaptor protein for downstream signaling [22].

Ligand binding to the N-terminal LRR-containing domain of TLRs initiates their homodimerization with an exception of TLR8 that is shown to be preexisting as an inactive homodimer [23,24,25]. The TIR domains of dimerized TLRs then recruit and interact with MyD88 [26]. This interaction provokes the assembly of “Myddosome”, a receptor proximal protein complex that results from death domain (DD) homotypic interactions between MyD88 and IL-1R-associated kinases (IRAK) 1 and 4 [27]. This is followed by the activation of IRAK1 by IRAK4. The activated IRAK1 then engages ubiquitin ligase, TNF receptor-associated factor 6 (TRAF6), which consequently activates TGFβ-activated kinase 1 (TAK1) by ubiquitination. Activated TAK1 then activates multiple signaling pathways: nuclear factor (NF)-κB activation by phosphorylation/activation of IKKβ of IKK complex; activation of mitogen-activated protein kinases (MAPKs) such as ERK1/2, p38, and JNK by phosphorylation, which consequently activates activator protein-1 (AP-1) family of transcription factors; activation of IFN-regulatory factor 7 (IRF7) in an IKKβ-dependent manner [28,29,30]. TLR3, however, signals exclusively via TRIF [31,32,33]. TRIF interacts with TRAF3 and TRAF6. The TRAF3 then recruits IKK complex and ubiquitinates NF-κB essential modulator (NEMO/IKKγ) resulting in its activation. The activated NEMO activates the kinase subunits of the IKK complex (IKKα and IKK β). The activated kinase subunits consequently activate TRAF Family Member-Associated NFKB Activator (TANK)-binding kinase 1 (TBK1) and inhibitor of nuclear factor kappa-B kinase subunit epsilons (IKKε, also known as IKKi), which drives phosphorylation/activation of IRF3 resulting in its nuclear translocation. The TRAF6, after interacting with TRIF, recruits and drives activation/ubiquitination of receptor-interacting protein kinase 1 (RIPK1), which activates TAK1 leading to activation and nuclear translocation of NFκB, IRF7, and AP-1 family of transriptioin factors [29]. The activation of NF-κB, AP-1 family of transcription factors, and various IRFs ultimately leads to the expression of type I and III IFNs along with proinflammatory cytokines, thus restricting viral propagation.

TLR3 is expressed by immune cells such as myeloid dendritic cells (mDCs) and macrophages as well as non-immune cells such as fibroblast and epithelial cells [22,34,35,36]. TLR3 in mDCs localizes in the early endosome, whereas macrophages, fibroblasts, and epithelial cells, in addition to the endosomal membrane, express TLR3 on the cell surface as well [22]. Nonetheless, TLR3-mediated signaling always initiates from the endosomal compartment with TLR3 activation sought to be relying on the uptake of extracellular virus-derived dsRNA molecule during phagocytosis of infected cells [22,37,38,39]. TLR3 binds to dsRNA (>40 bp) as a dimeric unit, with each monomer having two dsRNA binding sites [40,41].

Multiple studies have demonstrated the role of TLR3 in response to IAV infection. IAV infection induced activation of TLR3 in bronchial epithelial cells leading to the induction of proinflammatory cytokines IL-8 and IL-6 in an NF-κB dependent manner [42]. Intranasal pretreatment of mice with TLR3 ligand provided protection against H5N1 influenza virus and seasonal influenza virus infection [43]. However, the role of TLR3 in antiviral immunity during IAV infection is challenged by a study using TLR3^-/-^ mice. In comparison to the WT mice, TLR3^-/-^ mice produced significantly reduced amount of inflammatory cytokines/chemokines and had a survival advantage after IAV infection, suggesting an excessive TLR3 activation contributes to IAV pathogenesis [44]. In agreement with this study, inhibition of TLR3 activation by a single-strand oligonucleotide resulted in decreased IAV infection [45].

Although TLR3 is evidently involved in the host response against IAV, its physiological ligand however remains uncharacterized. The IAV is a single-stranded RNA virus, and the dsRNA intermediates formed during replication are believed to be destroyed by helicase, UAP56 [46]. However, Son et al. 2015 [47], by employing sensitive monoclonal antibodies along with protease treatment, showed that dsRNA is indeed present in the nucleus and cytoplasm of IAV infected cells [47]. In this scenario, it is highly likely that TLR3 is activated by uptake of such virus-derived dsRNA molecules during phagocytosis of infected cells by the surrounding immune and non-immune cells. 

TLR7 and TLR8 are highly homologous endosomal ssRNA sensors [48,49,50]. TLR7 is predominantly expressed in B cells and plasmacytoid DCs (pDCs) alongside respiratory epithelial cells, whereas TLR8 is expressed by human monocytes, macrophages, and mDCs [51,52,53]. Although both TLR 7 and TLR 8 recognize viral ssRNA, they differ much in the characteristics of ssRNA they bind to. Both possess two RNA binding sites in their LRR domain, the first binding site being specific for nucleosides, whereas the second binding site is specific for oligonucleotides. However, TLR 7 at site 1 prefers guanosine, whereas TLR 8 prefers uridine [24,54,55]. Similarly, TLR 7 at site 2 prefers a 3 nucleotide motif with U in the middle and TLR 8 whereas prefers UG [24,54,55]. 

Influenza virus is a ssRNA virus and it is, therefore, reasonable that TLR7/TLR8 recognizes the single-stranded genome of the virus inside endosomes. Wang et al. showed IAV RNA activates both TLR7 and TLR8 in human neutrophils, producing inflammatory cytokines MIP-1β and IL-8 after IAV infection. Using murine neutrophils isolated from TLR7 knockout mice, the author further proved that TLR7 is essential for IAV induced inflammatory cytokine production [56]. A most recent study reported in human monocytes showed that IAV infection activates TLR7 and TLR8. However, activation of TLR7 and TLR8 resulted in different signaling pathways and thus different phenotypes after IAV infection. In particular, TLR7 activation leads to increased expression of T_H_17 cell polarising cytokines, whereas TLR8 predominantly upregulated the expression of type 1 interferons and T_H_1 cell polarizing cytokines [57]. Notably, TLR7 has recently been reported to sense IAV in human platelets leading to C3 release. The released C3 then activate neutrophils causing them to undergo NETosis which may contribute to IAV mediated myocardial infarction [58]. 

TLR4 is expressed mainly on the surface of myeloid cells such as neutrophils, macrophages, and mDCs. It is generally activated by unique microbial cell wall components such as LPS [59]. Interestingly, TLR4 activation is also reported in influenza virus infection due to the release of endogenous DAMPs such as High Mobility Group Box1 (HMGB1), oxidized phospholipids, and Calgranulin B/MRP-14 from the infected cells [60,61,62]. Stimulation of TLR-4 by endogenous DAMPs leads to the induction of cytokines and interferons in Myd88- and TRIF-dependent manner. Consequently, treatment with TLR4 antagonist Eritoran has been reported to protect mice from influenza induced severe lung injury [63]. 

TLR10 is also reported to be involved in the innate immune response to IAV [64]. The study showed IAV infection upregulates TLR10 expression in human monocyte-derived macrophages and in a human monocytic cell line THP-1. Activation of TLR10 leads to an enhanced induction of cytokines such as IL-8, IL-6, IL-29, and IFN-β. Knockdown of TLR10 in THP-1 cells consequently showed considerably reduced cytokines expression after IAV infection. 

In all, TLRs are mainly expressed in immune cells. TLR ligands thus have the potential for therapeutic and vaccine interventions. Mice receiving lipd-derived nanoparticles containg TLR agonists had robust antiviral activity that inhibited IAV replication, and enhanced both humoral and cell-mediated responses when used as a vaccine adjuvant [65].

## 4. Retinoic Acid-Inducible Gene I (RIG-I)

RIG-I belongs to RIG-I-like receptor (RLR) family which are the key sensors of viral infection and induces expression of type I IFN and proinflammatory cytokines. The RLR family of proteins has three members: RIG-I, melanoma differentiation-associated protein 5 (MDA5), and laboratory of genetics and physiology 2 (LGP2). All three members share a central helicase domain and a C-terminal domain (CTD). The helicase (Hel) domain is further subdivided into three subdomains: Hel1, Hel2i, and Hel2. RIG-I and MDA5, however, have two additional N-terminal tandem caspase activation and recruitment domain (CARDs). The helicase domain and CTD are responsible for binding to the immunostimulatory RNA; the CARDs domain mediates the downstream signal transduction. Lack of CARDs essentially makes LGP2 signaling incompetent and it is believed to have a role in the regulation of RIG-I and MDA5. RLR family members are primarily cytosolic except for RIG-I, a small fraction of which localizes to the nucleus as well and is reported to sense nuclear replicating IAV [66,67]. Although both RIG-I and MDA5 recognize viral dsRNA, RIG-I specifically recognizes short dsRNA of ~10 to 19 bp, whereas MDA5 recognizes relatively longer viral dsRNA [68,69,70].

**Figure 2:**
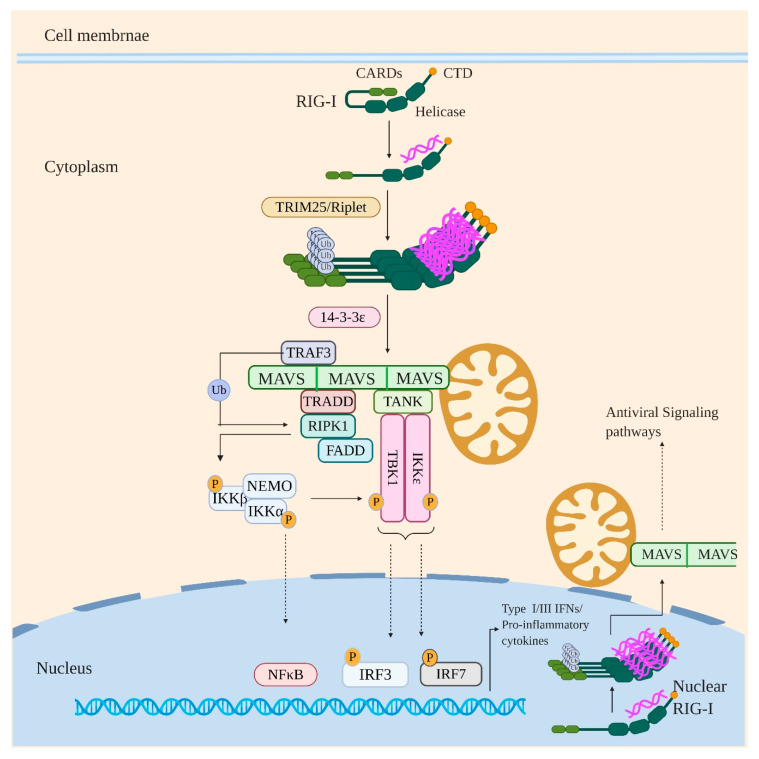
RIG-I signaling in response to viral infection. Under sterile conditions, RIG-I is generally present in closed conformation with the CARD2 domain interacting with the Helicase domain. However, on binding to immunostimulatory RNA, the CARDs are released and undergo K63Ub by ubiquitin ligases such as TRIM25 and Riplet. This drives RIG-I oligomerization and interaction with MAVS which leads to MAVS activation and oligomerization into MAVS filaments. Activated/oligomerized MAVS then interacts with TRAF3 and TRADD which itself exists in a complex with RIPK1 and FADD. The TRAF3 then activates RIPK1 by polyubiquitination which in turn activates IKK kinase complex by phosphorylation leading to activation and nuclear translocation of NF-κB. The activated IKK complex also activates TBK1/IKKε by phosphorylation which results in activation and nuclear translocation of IRF3 and IRF7. Signaling via this axis eventually induces the production of IFNs and proinflammatory cytokines. Notably, the nuclear resident RIG-I after recognizing the viral RNA in the nucleus is proposed to undergo oligomerization in the nucleus itself and interact with MAVS in the regions of proximity between the nuclear and mitochondrial membrane and thus inducing the antiviral signaling pathways.

RIG-I is expressed in all cell types but is shown to be crucial for viral detection in infected epithelial cells, alveolar macrophages, and conventional DCs [71]. RIG-I recognizes short blunt dsRNA with a 5′ di- or triphosphate with the 5′ terminal nucleotide unmethylated at 2’O position of ribose sugar [68,69,72,73,74]. The CTD of RIG-I possesses a binding pocket for 5’ di/triphosphate and also accommodates the unmethylated 2’O group of 5’ terminal nucleotide [75,76,77]. The helicase domain, on the other hand, makes extensive contacts with the base-paired region of dsRNA, whereas the N-terminal CARDs mediates downstream signaling (Figure 2) [70,78,79].

Under homeostatic conditions, RIG-I is present in a signaling incompetent closed conformation with its CARDs sequestered by specific contact between CARD2 and Hel2i subdomain of the central helicase domain [78]. Binding to immunostimulatory RNA via helicase and CTD drives large conformational changes in RIG-I which liberates the CARDs from helicase domain to interact with downstream signaling adaptors (Figure 2). Once liberated, the CARD2 is subjected to K63 linked polyubiquitination (K63Ub) by various ubiquitin ligases such as TRIM25, Riplet, TRIM4, and Mex-3 RNA binding family member C (MEX3C) [80,81,82,83]. Previous study showed Riplet is indispensible for TRIM25 to activate RIG-I signaling [84]. Recently, Hyman et al. demonstrated that deletion of *Trim25* does not have any impact on IFN response to some RNA viruses infection, including IAV, influenza B virus, or sendai virus. In an endogenous setting, the study suggests that Riplet, and not TRIM25, is required for the ubiquitination of RIG-I. However, this does not exclude the antiviral role of TRIM25, possibly through another mechanism [85]. The K63Ub drives RIG-I oligomerization by stabilizing RIG-I CARDs in signaling competent, oligomeric, “lock–washer” conformation formed of 2CARDs tetramer [86,87]. RIG-I in this oligomerized/signaling primed state interacts with 14-3-3ε protein which mediates its translocation to adaptor protein MAVS (mitochondrial antiviral signaling protein) [88]. MAVS localizes primarily to the mitochondrial outer membrane (MOM) but is also found on mitochondrial-associated membranes (MAMs) such as the endoplasmic reticulum and in the membrane of peroxisomes [89,90,91]. MAVS and RIG-I interact homotypically with each other via their CARD domains, which results in the activation and oligomerization of MAVS to form MAVS filaments. Activated/oligomerized MAVS subsequently interacts with TRADD (TNFR-associated death domain protein) via DD-DD homotypic interactions [92]. TRADD itself exists in a complex with FADD (FAS associated protein with death domain) and RIPK1 (Receptor-interacting serine/threonine-protein kinase 1); the complex being known as “TRADDosome”. MAVS also directly recruits TRAF3 which ubiquitinates RIPK1. The ubiquitinated RIPK1 recruits NEMO which results in activation of RIPK1 kinase activity. The activated RIPK1 phosphorylates/activates IKKβ and IKKα subunit of IKK complex, resulting in NF-κB induced gene expression. MAVS also directly interacts with TANK which is interacting with TBK1 and IKKε. The activated IKK complex aslo activates TBK1 and IKKε, which in turn activates IRFs (3 and 7) resulting in their nuclear translocation [93,94]. RIG-I induced signaling thus finally culminates with the production of IFNs (Types I and III) and proinflammatory cytokines.

Innate immune sensing of the influenza virus except pDCs is strictly dependent on RIG- I. IAV infection generates a variety of RIG-I agonists: the most critical RIG-I agonist being the viral genome itself [95]. Consistent with this, Liu et al. showed that IAV vRNA activates RIG-I by the genomic panhandle: a short double-stranded region (~16 bp) formed due to self-complementarity between the 5’ and 3’ ends of viral genomic segments. The study reported IFN stimulation by direct binding of the panhandle region to RIG-I; the coding region of the genome being inessential for RIG-I stimulation [96]. In addition to the intact viral genome, the incoming viral defective interfering (DI) genome also contributes to RIG-I activation in a panhandle dependent manner [97,98]. Recent studies indicate that mini viral RNA (mvRNA), a panhandle-forming shorter than 80 nts DI-like RNA, also activates RIG-I. The formation of mvRNAs is attributed to a faulty viral polymerase and an imbalanced polymerase to NP ratio [99]. Further, two distinct aberrant RNAs representing abortive replication products were detected in NP free reconstitution [98]. Addition of NEP in NP-free reconstitution enhanced synthesis of small viral RNA (svRNA) that was previously shown to be non-immunostimulatory by itself upon transfection [100,101]. Interestingly, the RNAs synthesized in NP-free reconstitutuion are immunostimulatory for RIG-I pathway, leading to IFN production [98]. Given the single strandedness of aberrant and svRNAs, their RIG-I activating potential might lie in their ability to form intermolecular duplexes with vRNA or cRNA [67].

RIG-I is previously acknowledged to be exclusively in the cytoplasm; recently, Liu et al. showed a genuine presence of RIG-I in the nucleus as well [66]. This nuclear resident RIG-I binds to IAV vRNPs in the nucleus and thus senses the nuclear replicating IAV genome, inducing antiviral immunity. Interestingly, after activation, the nuclear RIG-I signals through the canonical signaling pathway that requires the involvement of cytoplasmic MAVS. The study proposed that the interaction between nuclear RIG-I and cytoplasmic MAVS may occur in the perinuclear regions where compromised nuclear membrane allows RIG-I and MAVS in close contact, leading to the activation of antiviral signaling pathways.

RIG-I signaling plays a pivotal role in restricing IAV replication. Mice deficient in the RIG-I-MAVS pathway showed delayed IAV clearance and decreased polyfunctional T cell responses against IAV. Mechanistically, RIG-I signaling reduces IAV infection through producing IFN and regulating host adaptive immune responses [102].

Given the antiviral role of RIG-I signaling, RIG-I stimulating RNAs have been developed as influenza therapeutics. Lin et al. designed a short dsRNA that possesses dual functions: an siRNA targeting IAV NP gene and an agonist for RIG-I activation. Compared to the single-functioned siRNA, the dual functional dsRNA could potently inhibit IAV infection in tissue culture and in mice [103]. Consistently, Coch and colleagues showed that a single low dosage injection of mice with RIG-I ligand confers protection from lethal IAV challenge for 7 days [104].

## 5. Z-DNA Binding Protein 1 (ZBP1)

ZBP1, an upcoming star of the innate immunity, is known to regulate cell death and inflammation in conditions varying from viral infection to embryonic development [105]. The story of ZBP-1 began in 1999 when it was identified as a novel gene upregulated in tumor stroma and activated macrophages and was called DLM-1 [106]. This was followed by identification of the N-terminal Z-DNA binding (Zα) domain in DLM-1 and crystallization of Zα domain with Z-DNA, a left-handed double-stranded DNA helix [107,108,109]. Hereafter, DLM-1 came to be known as Z-DNA binding protein 1. Taniguchi and colleagues in 2007 reported ZBP-1 as an innate sensor of viral DNA while proposing ZBP-1 another name: DNA-dependent activator of IFN-regulatory factors or DAI [110]. Another twist in the tale came when ZBP1 was found to be the fourth mammalian protein harboring receptor-interacting protein homotypic interaction motif (RHIM) domains similar to RHIM domains of RIPK1, RIPK3, and TRIF that paved the way for current role of ZBP1 in cell death and inflammation [111,112].

Structurally, ZBP1 is composed of two N-terminal Z-DNA binding (Zα1 and 2) domains, two central RHIM domains, and a conserved C-terminal domain (CTD). The Zα2 domain is reported to bind Z-DNA and Z-RNA [109,113,114,115,116,117]. The RHIM domains mediate ZBP1 dependent cell death and inflammatory responses via interaction with other RHIM domain-containing proteins such as RIPK1 and RIPK3 [111,112]; CTD is responsible for type I IFN induction in response to immunostimulatory DNA [110]. ZBP1 is primarily a cytosolic protein but it also has been recovered from the nucleus in an infected environment [117,118,119,120].

**Figure 3:**
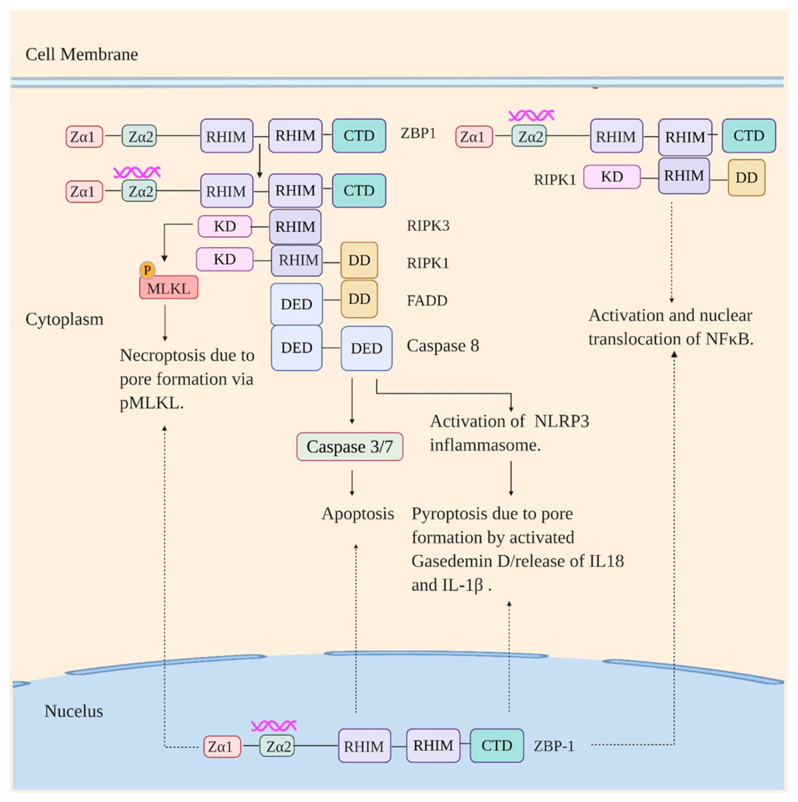
ZBP1 signaling in response to viral infection. ZBP1 on recognizing viral RNA in the cytoplasm interacts with RIPK1 and RIPK3 via their RHIM domains. Signaling via the ZBP1-RIPK1 axis results in activation and nuclear translocation of NF-κB. Signaling via ZBP1-RIPK3 axis, in turn, drives necroptosis via phosphorylation of MLKL by RIPK3 and apoptosis and NLRP3 inflammasome activation (thus pyroptosis) via ZBP1-RIPK3-RIPK1-FADD-Casapse8 axis (RIPK3 interacts with RIPK1 via RHIM domain which in turn interacts with FADD via death domain (DD), which subsequently interacts with Caspase 8 via death effector domain (DED)). KD: Kinase domain. The pathways are also initiated on the recognition of immunostimulatory RNA by ZBP1 in the nucleus.

Human ZBP1 is predominantly expressed in lymphatic tissues including lymph node, peripheral leukocytes, splenic cells, tonsils, and bone marrow and to a lesser extent in thymus, lung, and liver [121]. Activation of ZBP1 by immunostimulatory DNA and RNA drives different signaling pathways resulting in different responses. ZBP1 when activated by immunostimulatory DNA induces type I interferon production via TBK1-IRF3 axis mediated by its CTD [110]. Stimulation by RNA ligand on the other hand leads to the RHIM domain-dependent association of ZBP1 with RIPK3 and RIPK1 (Figure 3). This is followed by the initiation of apoptosis (a non-inflammatory programmed cell death mediated by executioner caspases [122]) via ZBP1-RIPK3-RIPK1-FADD (Fas-associated protein with death domain)-caspase-8 axis and necroptosis, programmed necrosis mediated by RIPK3 and MLKL (Mixed-lineage kinase domain-like protein) [123], via ZBP1-RIPK3-MLKL axis [115,117,124]. The ZBP1-RIPK3-RIPK1-FADD-Caspase 8 axis also parallelly regulates activation of NLRP3 inflammasome [118,125]. The NLRP3 inflammasome, which will be discussed in detail below, is a multiprotein complex comprising of NLRP3, apoptosis-associated speck-like protein (ASC), NIMA-related kinase 7 (NEK7), and Caspase 1, and its activation leads to cell death by pyroptosis (inflammasome-dependent cell death mediated by inflammatory caspases and gasderminD (GSDMD) [94,126,127]) along with the secretion of proinflammatory cytokines IL-1β and IL-18. ZBP1-RIPK1 axis on the other hand activates NF-κB leading to the induction of proinflammatory cytokines [118].

ZBP1 is a recently identified innate sensor of IAV and lies at the helm of various IAV-induced programmed cell death pathways, inflammasome activation, and production of proinflammatory cytokines during IAV infection. Consistent with this, Thapa et al. reported that ZBP1^-/-^ mice succumbed to IAV infection and displayed a significant delay in viral clearance in comparison to the WT mice [115]. Recently, Zhang and colleagues also reported greater mortality in ZBP1^-/-^ IAV infected mice likely due to their inability to control pulmonary viral spread [117]. Although being involved in responses against IAV there has not been much consensus reached on IAV ligand for ZBP1. The initial study by Kuriakose reported that ZBP1 recognizes IAV NP and polymerase subunit PB1 [118]. The recognition of the viral proteins by ZBP1 was reported to provoke multiple responses in the IAV infected cells: initiation of apoptosis and necroptosis mediated by RIPK3, NF-κB activation via ZBP1-RIPK1 axis, and activation of NLRP3 inflammasome via RIPK3-Caspase8 axis. Kesvardhana et al. then reported ZBP1 interaction with IAV vRNP which induces programmed cell death [128]. Thapa et al. reported that ZBP1 recognizes IAV genomic RNA following nuclear export and suggested the genomic RNA either being present in Z-RNA like conformation or adopting a Z-conformation on binding to ZBP1. Sensing of the genomic RNA was reported to lead to RIPK3-dependent apoptosis mediated by RIPK1 and necroptosis mediated by MLKL [115]. The RIPK3-dependent aopotosis and necropotosis in response to ZBP1 activation during IAV infection was also reported by Nogusa et al. [124]. Interestingly, Zhang and colleagues have recently shown that replicating IAV produces Z-RNAs in the nucleus of infected cells which are recognized by ZBP1 resulting in necroptosis via ZBP1-RIPK3-MLKL axis [117]. Therefore, the current view holds that ZBP1 (in nucleus as well as cytoplasm) is activated by Z-RNAs, which results either directly from IAV replication or transition from A to Z conformation on binding to ZBP1. Activation of ZBP1 is then followed by various forms of programmed cell death, inflammasome activation, and induction of proinflammatory cytokines.

## 6. NOD-, LRR-, and Pyrin Domain-Containing Protein 3 (NLRP3)

NLRP3 belongs to the nucleotide-binding oligomerization domain (NOD)-like receptors (NLRs) family and specifically to the NLR pyrin domain-containing (NLRP) subfamily of NLRs. The human NLR family has 23 identified members with 14 members shared by the NLRP subfamily (NLRP 1-14). The NLR family members are primarily cytosolic and regulate inflammation and programmed cell death in response to cellular stress. All the NLR family members share an N-terminal effector domain, a central nucleotide-binding and oligomerization (NOD) domain (also known as NACHT domain), and a C-terminal LRR domain [129]. The N-terminal region varies between various members, which results in the induction of diverse signaling pathways; the NLRP family contains an N-terminal pyrin domain (PYD). The central NACHT/NOD domain has ATPase activity which is vital for NLRs oligomerization and function, the C-terminal LRR domain is responsible for ligand binding while the N-terminal region provides effector function by interacting with downstream signaling adaptors in a homotypic manner [130]. Before activation, the NLRs are present in a monomeric form with the LRR domain folding back into the NOD/NACHT domain and are defined to be auto-suppressed [131].

**Figure 4:**
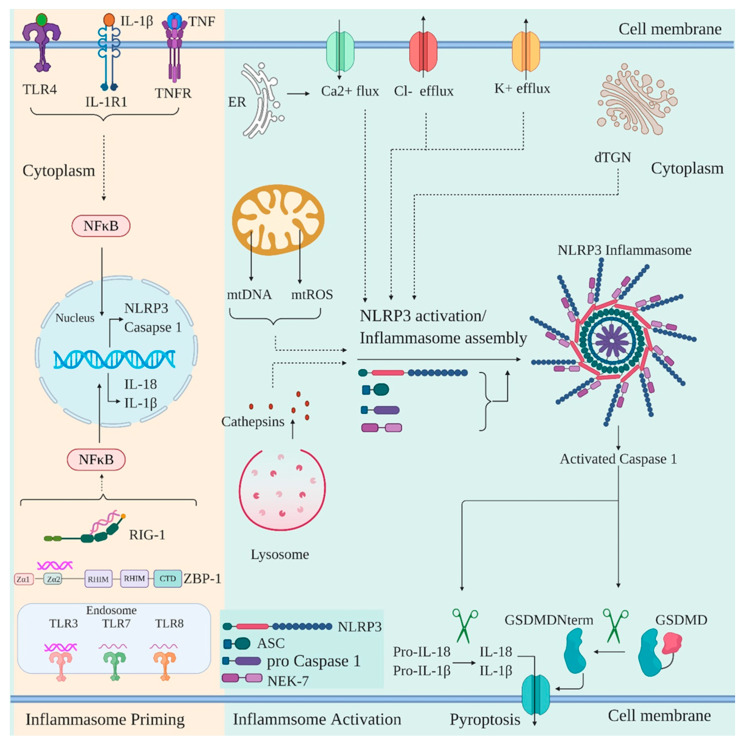
Priming and activation of NLRP3 inflammasome in response to viral infection. NLRP3 inflammasome in response to viral infection is primed by the cytokines such as TNFα and IL-1β or through viral recognition by PRRs such as RIG-I, TLRs, and ZBP-1. The signals converge at NF-κB activation and nuclear translocation leading to the gene transcription of NLPR3, caspase 1, IL-1β, and IL-18. The priming is followed by the activation of the inflammasome in response to a variety of upstream signals such as K^+^/Cl^−^ efflux and Ca^2+^ Flux, either due to the opening of plasma membrane channels or release from the endoplasmic reticulum (ER). Mitochondrial disruption and release of mtDNA and mtROS into the cytoplasm, lysosomal rupture and release of cathepsins, dispersion of *trans* Golgi network (dTGN) can also activate inflammasome. The activated inflammasome leads to the cleavage of pro-caspase 1 to generate activated form of caspase 1, which then cleaves pro-IL-1β and pro-IL-18 to their mature form of IL-1β and IL-18. Active caspase 1 also cleaves GSDMD into a 31 kDa N-terminal fragment (GSDMDNterm) and a 22 kDa C-terminal fragment. GSDMDNterm then permeabilizes the plasma membrane and induces pyroptosis.

NLRP3 is a tripartite protein that contains an N-terminal PYD domain, central NOD/NACHT domain, and C-terminal LRR domain. NLRP3 activation is widely known to form an inflammasome, a cytosolic multiprotein complex that is comprised of oligomerized NLRP3, ASC, and caspase-1 [132]. The NLRP3 inflammasome assembly and activation is achieved by two steps: priming of the inflammasome and activation of the inflammasome. The priming of the inflammasome involves the upregulation of the expression of inflammasome components (NLRP3, caspase 1, pro-IL-1β, and pro-IL18). This is achieved by NF-κB activation which is induced either by cytokines such as TNFα and IL-1β or by recognition of various DAMPs and PAMPs by PRRs such as TLRs, RIG-1, NOD2, and ZBP1 (Figure 4) [133,134,135]. Priming of the inflammasome is followed by activation of NLRP3 by a wide variety of upstream signals consequential to cellular stress posed by the presence of various pathogens. Upon activation, NLRP3 oligomerizes via NACHT-NACHT homotypic interaction. Oligomerized NLRP3 recruits ASC via PYD-PYD interaction and provides a nucleation site for ASC filaments formation via PYD-PYD interaction between the ASC molecules. This is followed by ASC filaments coalescing into a micrometer-sized “ASC speck” which is a hallmark of inflammasome activation [136,137,138]. The ASC speck recruits pro-caspase 1 via CARD-CARD interaction resulting in proximity induced self–cleavage of pro-caspase 1 [139]. The activated caspase 1 proteolytically processes and activates pro-IL-1β, pro-IL-18, and the pyroptotic factor GSDMD [94,140,141]. Both IL-1β and IL-18 subsequently induce an adaptive immune response against pathogens whereas the activated GSDMD induces cell death by pyroptosis [142,143,144]. Of note, NEK7 is also shown to oligomerize specifically with activated NLRP3 into a complex which is essential for ASC speck formation and caspase 1 activation and thus appears to be an integral part of NLRP3 inflammasome [145,146].

Independent studies have identified various inflammasome activating upstream signals. Ionic imbalances due to K^+^ / Cl^−^ efflux and Ca^2+^ mobilization into the cell either via the opening of plasma membrane channel or release of Ca^2+^ into the cytosol from ER are an important upstream signal for NLRP3 activation [147,148,149,150,151]. Additionally, mitochondrial (mt) damage and thus release of mtROS / mtDNA into the cytoplasm is another crucial signal for NLRP3 activation [152,153,154,155,156]. Recently, disassembly of trans-Golgi network (TGN) to vesicles called dispersed TGN (dTGN) has been reported to be another mechanism for NLRP3 activation. The phospholipid phosphatidylinositol-4-phosphate (PtdIns4P) on dTGN is implicated in recruiting NLRP3 and promoting its oligomerization [157]. In addition to the DAMPs described above, NLRP3 is also able to sense the presence of PAMPs via various PRRs. PRRs, such as DExD/H-box RNA helicase family members DHX33 and DDX19A, are reported to interact with and activate NLRP3 after sensing reoviral genomic RNA [158,159]. Similarly, the roles of ZBP1, RIG-I, and TLR3 have also been captured in driving activation of NLRP3 inflammasome [160].

Various studies have demonstrated the criticality of NLPRP3 inflammasome activation during IAV infection. Accordingly, Thomas et al. demonstrated that NLRP3^-/-^ and caspase 1^-/-^ mice were highly susceptible to both low and high dosage of IAV resulting in enhanced morbidity [161]. Similarly, Allen et al. also showed that mice deficient in NLRP3, ASC, and Caspase 1 displayed a marked increase in mortality and dampened immune response to IAV [162]. Mechanisms such as ionic imbalances, lysosomal rupture, mitochondrial disruption, and ROS generation seem to be governing NLRP3 activation in IAV-infected cells. Consistent with this, the mutant influenza virus lacking H^+^ transport activity of M2 protein failed to induce IL-1β secretion from bone marrow-derived macrophages (BMDMs) and bone marrow-derived dendritic cells (BMDCs) which was restored by ectopic expression of M2, suggesting the ion channel activity of M2 plays a role in inflammasome activation via mediating K^+^ efflux [163]. Furthermore, IAV NA is reported to disrupt lysosomes and release cathepsins into the cytosol, which was previously reported to disrupt mitochondrial membrane and release ROS in the cytoplasm leading to NLRP3 activation [164,165]. The influenza virus PB1-F2 is another protein correlated with inflammasome activation [166]. PB1-F2 during influenza virus infection localizes to the mitochondrial inner membrane (MIM) and attenuates mitochondrial membrane potential, thus significantly affecting mitochondrial dynamics which leads to mitochondrial disruption and accelerated ROS production which forms the basis of NLRP3 activation [167]. Similarly, Park et al. also reported mitochondrial fission mediated by the RIPK1-DRP1 signaling axis leads to NLRP3 inflammasome dependent IL-1β secretion from swine influenza virus-infected macrophages. The secretion of IL-1β from infected macrophages was found to be contingent upon accelerated mtROS production [168]. Conversely, the IFN-induced 2’,5’-oligoadenylate synthetase (OAS)/ribonuclease L (RNase L) system is also associated with NLRP3 activation as the release of IL-1β is minimal in IAV-infected RNase L-deficient BMDCs. The study also reported that RNase L-cleaved RNA facilitates the formation of a complex containing RNA helicase DHX33, MAVS, and NLRP3 which results in NLRP3 activation in a DHX33 dependent manner [169]. Interestingly, commensal bacteria are also reported to play an important role in regulating NLRP3 inflammasome activation in the lungs of influenza virus-infected mice. Ichinohe et al. reported reduced expression of IL-1β, IL-18, and NLRP3 during influenza virus infection of antibiotic-treated mice suggesting a role of commensal bacteria in the priming of inflammasome [170].

Notably, another NLR family member, NOD2 has also been implicated in sensing the ssRNA genome of IAV and interacting with MAVS which leads to type I IFN induction via IRF3 [171].

## 7. Influenza Virus: A Brilliant Strategist in Evading Innate Immune Recognition

The innate immune system as we know employs a myriad of mechanisms to subvert the influenza virus infection. The virus, however, has also evolved with time to counteract such responses. Below we discuss the viral evasion mechanisms mediated by the three major viral immunomodulators.

## 8. The Non-Structural Protein 1 (NS1)

IAV NS1 is encoded by genome segment 8 as a continuous primary transcript and expresses abundantly in infected cells. NS1 most often is a protein of 230 amino acids (AAs), but variations in length (219 to 237 AAs) are found in different strains. The NS1 is divided into two major domains: an N-terminal RNA binding domain (RBD, AA 1-73) and a C-terminal effector domain (ED, AA 88-202). The two domains are linked by a flexible linker region of 10-15 AAs, and the ED is followed by a short unstructured tail. The RBD mediates NS1 dimerization which is necessary for binding to dsRNA whereas the ED is known to interact with a myriad of host factors to exert various functions related to the modulation of viral replication and virulence [172].

IAV NS1 is well known for its anti-interferon activity. Replication of mutant IAVs lacking NS1 or expressing truncated NS1 is largely attenuated in IFN competent cells and in vivo [173,174]. RIG-I, as explained before, is a key player of innate antiviral responses to the influenza virus and is thus a prime target of NS1. NS1 inhibits RIG-I function through multiple mechanisms. Besides the initially identified mechanism by which NS1 sequesters RIG-I agonist dsRNA through its RBD, NS1 interacts directly with the RIG-I CARD domain, thus inhibiting RIG-I activation in a strain-specific manner [175,176,177]. NS1 also interacts with various key proteins involed in the RIG-I pathway, such as ubiquitin ligases TRIM25 and Riplet, and thus prevents RIG-I ubiquitination, oligomerization, and activation [178,179,180]. Structurally, binding of NS1 to TRIM25 displaces the PRYSPRY domain in TRIM25, thus interfering with RIG-I ubiquitination [180]. Additionally, NS1 is shown to upregulate expression of A20/tumor necrosis factor α-induced protein 3 (TNFAIP3), a cytoplasmic ubiquitin editing protein which is known to negatively affect RIG-I mediated activation of IRF3 [181,182].

The role of NS1 in antagonizing NLRP3 inflammasome activation is also documented. Park et al. showed NS1 derived from the 2009 pandemic strain significantly inhibits NLRP3 inflammasome-mediated IL-1β production in porcine macrophages in comparison to swine influenza virus strains. In mechanism, pandemic influenza NS1 suppresses the ASC ubiquitination on lysine residues K110 and K140, leading to impaired ASC speck formation and NLRP3 inflammasome activation [183]. Similarly, Moriyama also observed NS1 protein inhibits NLRP3 inflammasome mediated IL-1β production by direct interaction with NLRP3. Furthermore, AA 38 and 41 in RBD and AA 96 and 97 in the TRIM 25 binding domain within NS1 are responsible for the suppression [184].

Necroptosis, a programmed cell death, constitutes an important aspect of host immune responses through the regulation of inflammation. Gaba et al. reported that IAV infection induces necroptosis in macrophages and epithelial cells; the NS1 protein of IAV interacts with MLKL through the coiled-coil domain 2 of MLKL. The interaction results in the increased MLKL oligomerization and membrane translocation. Moreover, the interaction enhances MLKL-mediated NLRP3 inflammasome activation, possibly via disrupted ionic homeostatsis, leading to increased IL-1β production [185].

Additionally, IAV NS1 employs other mechanisms to inhibit innate immune signaling. NS1 binds and blocks IKKβ, thus inhibiting activation of NF-κB and expression of antiviral genes [186,187,188]. NS1 inhibits host gene expression by binding to cleavage and polyadenylation specificity factor (CPSF) [189], splicing [190,191], and nuclear export factors [192], which in turn also affects IFN production and ISGs induction.

## 9. PB1-F2

PB1-F2 is an auxiliary protein encoded from a + 1 ORF of PB1 gene [193,194]. The avian IAVs generally express full-length PB1-F2 of 90 AAs, while the mammalian IAVs produce truncated PB1-F2 (≤78 aa) due to a premature stop codon [195,196]. IAV PB1-F2 has been reported to suppress IFN and cytokine response in infected cells through various mechanisms. It is known that mitochondrial membrane potential (Δ*ψ*_m_), a central mitochondrial phenomenon, is cardinal to the activation of both RLR pathways and the NLRP3 inflammasome during IAV infection [197,198]. An enthralling aspect of these findings is that Δψm across the MIM and the assembly of supramolecular signaling complexes on mitochondrial outer membrane (MOM) platform are coupled and activates the immune response concertedly. Consistently, PB1-F2 translocates to the mitochondrial inner membrane space in a Tom40 import channel-dependent manner and accelerates mitochondrial fragmentation leading to attenuation of Δ*ψ*_m_ [167]. This suppresses the activation of both RLR signaling and the NLRP3 inflammasome, thus compromising the cellular innate immune response.

PB1-F2 via its C-terminal region interacts directly with the transmembrane region of MAVS and dissipates Δψm, which is essential for MAVS-mediated interferon signaling [199,200]. Notably, the transmembrane domain of MAVS is responsible for its oligomerization which provides a docking platform for downstream signaling molecules and PB1-F2 association with this domain apart from the dissipation of Δψm might also hinder MAVS oligomerization and thus the IFN signaling. Additionally, the interaction of PB1-F2 with IRF3 has also been correlated to reduced levels of IFN-β [201]. Interestingly, Gloire et al. reported a significant reduction in NF-κB binding to its target genes in IKKβ^-/-^ cells and suggested a role of IKKβ in driving expression of NF-κB genes in the nucleus in an unknown manner [202]. Consistent with this, full-length PB1-F2 interacts with IKKβ, and this interaction severely impairs NF-κB binding to its target genes [203]. Further, Leymaire et al. reported interaction between PB1-F2 and cellular calcium-binding and coiled-coil domain 2 (CALCOCO2/NDP52) which negatively affected TBK1 induced signaling pathways (IRF3/IRF7 activation) [204]. This stems from the ability of CALCOCO2 to interact with TBK1 adaptor proteins, NAP1 and SINTBAD, and thus contribute to the functional assembly of TBK1 [205].

## 10. PA-X

PA-X is another IAV auxiliary protein which is encoded by the PA gene. PA-X shares the N- terminal 191 AAs with PA protein. However, the ribosome shift occurs at the UUUCGU region, resulting in the synthesis of the rest of PA-X AAs from +1 ORF. Most human IAV strains produce 252 AAs PA-X with a C-terminal extension of 60 AAs [206,207]. PA-X is mainly known to shut off host translation by selective degradation of host Pol II transcripts via its N-terminal endonucleolytic domain in coordination with the host 5′ to 3′-exonuclease Xrn, thus affecting the innate immune response [208,209,210,211]. The targeted degradation of host transcripts by PA-X is achieved by its interaction with the host pre-mRNA processing proteins (CPSF5/6) that modifies the 3’ end of the nascent transcript and thus recruits PA-X to these nascent host mRNAs, fostering their degradation by PA-X in the nucleus [212]. Interestingly, PA-X, besides degrading host mRNA, has also been hypothesized to degrade viral dsRNA to evade recognition by various PRRs as PA-X mutated IAV produces much higher amounts of IFN-β than the wild type [213]. PA-X also prevents type I IFN production through the RIG-I-MAVS pathway. Using a PA-X-deficient virus in the background of PR8 strain, Rigby et al. recently showed mRNA levels of *Ifna4* and *Ifnb1* were elevated in the lungs of mice infected with PA-X deficient virus than in the mice infected with the WT virus. Moreover, the expressions of *Ifnb1* and *Ifna4* are comparable in the lungs of *Mavs*^−/−^ mice infected with WT virus or PA-X-deficient virus, suggesting PA-X inhibiton of the expression of *Ifnb1* and *Ifna4* is through a MAVS-dependent manner [214]. In addition to the above-mentioned three major viral immunomodulators, other mechanisms of innate immune evasion are also revealed. The polymorphism of PB2 at position 627 is identified as a factor to modulate RIG-I sensing. Compared to vRNP containing mammalianized PB2-627K, vRNP containing avianized PB2-627E has increased RIG-I recognition. This was attributable to the lower affinity of PB2-627E to NP, which may enable RIG-I to a better access to the nucleocapsid-associated panhandle RNA. Thus, mammalian adapted PB2-627K is thought to be an viral evasion strategy to avoid RIG-I sensing [215]. Previously, the nuclear replicating feature of IAV was reagarded as an immune evasion strategy to avoid cytoplasmic RIG-I sensing. Since the discovery of nuclear RIG-I and ZBP1, which can recognized viral RNA in the nucleus and trigger appropriate antiviral responses production, this compartmentized evasion strategy seems to be less efficient.

## 11. Negative Host Regulators

Inflammation as we know is a double-edged sword and can be malicious to the host if not regulated. Accordingly, the host has evolved to balance the proinflammatory pathways in a manner to contribute to effective viral clearance without inflicting damage to itself, with various regulators to play a role in it. Consistent with this, Huang et al. reported the transcription factor peroxisome proliferator-activated receptor gamma (PPAR-γ) as a negative regulator of inflammation during IAV infection in alveolar macrophages (AM) as PPAR-γ deficiency in AM enhanced pulmonary inflammation and host morbidity [216]. Similarly, transporter 1, ATP-binding cassette, subfamily B (TAP1) is shown to be upregulated upon IAV infection and inhibit NF-κB-mediated proinflammatory cytokine production by targeting the TAK1 complex [217]. Furthermore, the NLR family protein NLRX1 is also reported to attenuate IAV induced inflammation by meddling with the RIG-I-MAVS signaling pathway and TRAF6 ubiquitin ligase, an essential component of antiviral TLR signaling [218]. Consequently, NLRX1^-/-^ IAV-infected mice displayed increased morbidity and marked histopathology. Moreover, LGP2, a member of the RLR family of receptors, is shown to negatively regulate the RIG-I signaling pathway during IAV infection. Consequently, transgenic mice overexpressing LGP2 displayed reduced inflammation during IAV infection with lower levels of IFNβ-mRNA, IFN-α, and TNF-α in their BAL fluid, and the mice had a significant survival advantage [219].

## 12. Conclusion Remarks and Future Perspective

Recent research has advanced our knowledge on how IAV infection is sensed by various PRRs and how the sensing triggers the signaling pathways, leading to the host innate immune responses. A large body of literature is also available towards understanding how IAV utilizes various mechanisms to counteract the host immune recognition and antiviral responses. However, many knowledge gaps remain to be filled. In particular, the identification of viral RNA species generated during IAV life cycle and their contribution to various PRRs’ activation warrant further investigation. With the identification of the nuclear sensors, we still do not know how the signal that initiates in the nuclus is transduced to the cytoplasm and being executed. Furthermore, RIG-I being found to reside in both nucleus and cytoplasm, it is important to understand how the two pools of RIG-I co-ordinately modulate IAV infection. Questions also remain open concerning how different PRRs work synergistically in vivo to respond and control IAV infection. Only with these questions answered would better approaches to control and prevent influenza infection be achieved.

## References

[1] Iwasaki A., Medzhitov R. (2015). Control of adaptive immunity by the innate immune system. Nat. Immunol..

[2] Amarante-Mendes G.P., Adjemian S., Branco L.M., Zanetti L.C., Weinlich R., Bortoluci K.R. (2018). Pattern Recognition Receptors and the Host Cell Death Molecular Machinery. Front. Immunol..

[3] Mostafa A., Abdelwhab E.M., Mettenleiter T.C., Pleschka S. (2018). Zoonotic Potential of Influenza A Viruses: A Comprehensive Overview. Viruses.

[4] Eisfeld A.J., Neumann G., Kawaoka Y. (2015). At the centre: Influenza A virus ribonucleoproteins. Nat. Rev. Microbiol..

[5] De Vries E., Tscherne D.M., Wienholts M.J., Cobos-Jiménez V., Scholte F., García-Sastre A., Rottier P.J.M., de Haan C.A.M. (2011). Dissection of the Influenza A Virus Endocytic Routes Reveals Macropinocytosis as an Alternative Entry Pathway. PLoS Pathog..

[6] Rust M.J., Lakadamyali M., Zhang F., Zhuang X. (2004). Assembly of endocytic machinery around individual influenza viruses during viral entry. Nat. Struct. Mol. Biol..

[7] Pinto L.H., Lamb R.A. (2006). The M2 proton channels of influenza A and B viruses. J. Biol. Chem..

[8] White J., Helenius A., Gething M.-J. (1982). Haemagglutinin of influenza virus expressed from a cloned gene promotes membrane fusion. Nature.

[9] Wu W.W.H., Sun Y.-H.B., Panté N. (2007). Nuclear import of influenza A viral ribonucleoprotein complexes is mediated by two nuclear localization sequences on viral nucleoprotein. Virol. J..

[10] Martin K., Helenius A. (1991). Transport of incoming influenza virus nucleocapsids into the nucleus. J. Virol..

[11] O’Neill R.E., Jaskunas R., Blobel G., Palese P., Moroianu J. (1995). Nuclear import of influenza virus RNA can be mediated by viral nucleoprotein and transport factors required for protein import. J. Biol. Chem..

[12] Wang P., Palese P., O’Neill R.E. (1997). The NPI-1/NPI-3 (karyopherin alpha) binding site on the influenza a virus nucleoprotein NP is a nonconventional nuclear localization signal. J. Virol..

[13] Cros J.F., García-Sastre A., Palese P. (2005). An unconventional NLS is critical for the nuclear import of the influenza A virus nucleoprotein and ribonucleoprotein. Traffic.

[14] Wu W.W., Weaver L.L., Panté N. (2007). Ultrastructural analysis of the nuclear localization sequences on influenza A ribonucleoprotein complexes. J. Mol. Biol..

[15] Huang S., Chen J., Chen Q., Wang H., Yao Y., Chen J., Chen Z. (2013). A second CRM1-dependent nuclear export signal in the influenza A virus NS2 protein contributes to the nuclear export of viral ribonucleoproteins. J. Virol..

[16] Shimizu T., Takizawa N., Watanabe K., Nagata K., Kobayashi N. (2011). Crucial role of the influenza virus NS2 (NEP) C-terminal domain in M1 binding and nuclear export of vRNP. FEBS Lett..

[17] Akarsu H., Burmeister W.P., Petosa C., Petit I., Müller C.W., Ruigrok R.W., Baudin F. (2003). Crystal structure of the M1 protein-binding domain of the influenza A virus nuclear export protein (NEP/NS2). EMBO J..

[18] Abdoli A., Soleimanjahi H., Tavassoti Kheiri M., Jamali A., Jamaati A. (2013). Determining influenza virus shedding at different time points in madin-darby canine kidney cell line. Cell J..

[19] Henle W., Liu O.C. (1951). Studies on host-virus interactions in the chick embryo-influenza virus system. VI. Evidence for multiplicity reactivation of inactivated virus. J. Exp. Med..

[20] O’neill L.A., Golenbock D., Bowie A.G. (2013). The history of Toll-like receptors—Redefining innate immunity. Nat. Rev. Immunol..

[21] Jin M.S., Lee J.-O. (2008). Structures of the toll-like receptor family and its ligand complexes. Immunity.

[22] Matsumoto M., Seya T. (2008). TLR3: Interferon induction by double-stranded RNA including poly(I:C). Adv. Drug Deliv. Rev..

[23] Choe J., Kelker M.S., Wilson I.A. (2005). Crystal Structure of Human Toll-Like Receptor 3 (TLR3) Ectodomain. Science.

[24] Zhang Z., Ohto U., Shibata T., Krayukhina E., Taoka M., Yamauchi Y., Tanji H., Isobe T., Uchiyama S., Miyake K. (2016). Structural Analysis Reveals that Toll-like Receptor 7 Is a Dual Receptor for Guanosine and Single-Stranded RNA. Immunity.

[25] Tanji H., Ohto U., Shibata T., Miyake K., Shimizu T. (2013). Structural Reorganization of the Toll-Like Receptor 8 Dimer Induced by Agonistic Ligands. Science.

[26] Vyncke L., Bovijn C., Pauwels E., Van Acker T., Ruyssinck E., Burg E., Tavernier J., Peelman F. (2016). Reconstructing the TIR side of the Myddosome: A paradigm for TIR-TIR interactions. Structure.

[27] Gay N.J., Gangloff M., O’Neill L.A.J. (2011). What the Myddosome structure tells us about the initiation of innate immunity. Trends Immunol..

[28] Bergstrøm B., Aune M.H., Awuh J.A., Kojen J.F., Blix K.J., Ryan L., Flo T.H., Mollnes T.E., Espevik T., Stenvik J. (2015). TLR8 Senses Staphylococcus aureus RNA in Human Primary Monocytes and Macrophages and Induces IFN-β Production via a TAK1–IKKβ–IRF5 Signaling Pathway. J. Immunol..

[29] Kawai T., Akira S. (2010). The role of pattern-recognition receptors in innate immunity: Update on Toll-like receptors. Nat. Immunol..

[30] Takaoka A., Yanai H., Kondo S., Duncan G., Negishi H., Mizutani T., Kano S.-I., Honda K., Ohba Y., Mak T.W. (2005). Integral role of IRF-5 in the gene induction programme activated by Toll-like receptors. Nature.

[31] Hoebe K., Du X., Georgel P., Janssen E., Tabeta K., Kim S.O., Goode J., Lin P., Mann N., Mudd S. (2003). Identification of Lps2 as a key transducer of MyD88-independent TIR signalling. Nature.

[32] Oshiumi H., Matsumoto M., Funami K., Akazawa T., Seya T. (2003). TICAM-1, an adaptor molecule that participates in Toll-like receptor 3-mediated interferon-beta induction. Nat. Immunol..

[33] Yamamoto M., Sato S., Hemmi H., Hoshino K., Kaisho T., Sanjo H., Takeuchi O., Sugiyama M., Okabe M., Takeda K. (2003). Role of Adaptor TRIF in the MyD88-Independent Toll-Like Receptor Signaling Pathway. Science.

[34] Matsumoto M., Funami K., Tanabe M., Oshiumi H., Shingai M., Seto Y., Yamamoto A., Seya T. (2003). Subcellular localization of Toll-like receptor 3 in human dendritic cells. J. Immunol..

[35] Jongbloed S.L., Kassianos A.J., McDonald K.J., Clark G.J., Ju X., Angel C.E., Chen C.-J.J., Dunbar P.R., Wadley R.B., Jeet V. (2010). Human CD141+ (BDCA-3)+ dendritic cells (DCs) represent a unique myeloid DC subset that cross-presents necrotic cell antigens. J. Exp. Med..

[36] Jelinek I., Leonard J.N., Price G.E., Brown K.N., Meyer-Manlapat A., Goldsmith P.K., Wang Y., Venzon D., Epstein S.L., Segal D.M. (2011). TLR3-specific double-stranded RNA oligonucleotide adjuvants induce dendritic cell cross-presentation, CTL responses, and antiviral protection. J. Immunol..

[37] Tatematsu M., Nishikawa F., Seya T., Matsumoto M. (2013). Toll-like receptor 3 recognizes incomplete stem structures in single-stranded viral RNA. Nat. Commun..

[38] Karikó K., Ni H., Capodici J., Lamphier M., Weissman D. (2004). mRNA is an endogenous ligand for Toll-like receptor 3. J. Biol. Chem..

[39] Ebihara T., Shingai M., Matsumoto M., Wakita T., Seya T. (2008). Hepatitis C virus-infected hepatocytes extrinsically modulate dendritic cell maturation to activate T cells and natural killer cells. Hepatology.

[40] Liu L., Botos I., Wang Y., Leonard J.N., Shiloach J., Segal D.M., Davies D.R. (2008). Structural Basis of Toll-Like Receptor 3 Signaling with Double-Stranded RNA. Science.

[41] Botos I., Liu L., Wang Y., Segal D.M., Davies D.R. (2009). The toll-like receptor 3:dsRNA signaling complex. Biochim. Biophys. Acta.

[42] Le Goffic R., Pothlichet J., Vitour D., Fujita T., Meurs E., Chignard M., Si-Tahar M. (2007). Cutting Edge: Influenza A Virus Activates TLR3-Dependent Inflammatory and RIG-I-Dependent Antiviral Responses in Human Lung Epithelial Cells. J. Immunol..

[43] Wong J.P., Christopher M.E., Viswanathan S., Karpoff N., Dai X., Das D., Sun L.Q., Wang M., Salazar A.M. (2009). Activation of toll-like receptor signaling pathway for protection against influenza virus infection. Vaccine.

[44] Le Goffic R., Balloy V., Lagranderie M., Alexopoulou L., Escriou N., Flavell R., Chignard M., Si-Tahar M. (2006). Detrimental contribution of the Toll-like receptor (TLR)3 to influenza A virus-induced acute pneumonia. PLoS Pathog..

[45] Poux C., Dondalska A., Bergenstrahle J., Palsson S., Contreras V., Arasa C., Jarver P., Albert J., Busse D.C., LeGrand R. (2019). A Single-Stranded Oligonucleotide Inhibits Toll-Like Receptor 3 Activation and Reduces Influenza A (H1N1) Infection. Front. Immunol..

[46] Wisskirchen C., Ludersdorfer T.H., Müller D.A., Moritz E., Pavlovic J. (2011). The cellular RNA helicase UAP56 is required for prevention of double-stranded RNA formation during influenza A virus infection. J. Virol..

[47] Son K.N., Liang Z., Lipton H.L. (2015). Double-Stranded RNA Is Detected by Immunofluorescence Analysis in RNA and DNA Virus Infections, Including Those by Negative-Stranded RNA Viruses. J. Virol..

[48] Diebold S.S., Kaisho T., Hemmi H., Akira S., Reis e Sousa C. (2004). Innate Antiviral Responses by Means of TLR7-Mediated Recognition of Single-Stranded RNA. Science.

[49] Lund J.M., Alexopoulou L., Sato A., Karow M., Adams N.C., Gale N.W., Iwasaki A., Flavell R.A. (2004). Recognition of single-stranded RNA viruses by Toll-like receptor 7. Proc. Natl. Acad. Sci. USA.

[50] Heil F., Hemmi H., Hochrein H., Ampenberger F., Kirschning C., Akira S., Lipford G., Wagner H., Bauer S. (2004). Species-specific recognition of single-stranded RNA via toll-like receptor 7 and 8. Science.

[51] Fan H., Ren D., Hou Y. (2018). TLR7, a third signal for the robust generation of spontaneous germinal center B cells in systemic lupus erythematosus. Cell Mol. Immunol..

[52] Ioannidis I., Ye F., McNally B., Willette M., Flaño E. (2013). Toll-like receptor expression and induction of type I and type III interferons in primary airway epithelial cells. J. Virol..

[53] Gorden K.B., Gorski K.S., Gibson S.J., Kedl R.M., Kieper W.C., Qiu X., Tomai M.A., Alkan S.S., Vasilakos J.P. (2005). Synthetic TLR agonists reveal functional differences between human TLR7 and TLR8. J. Immunol..

[54] Zhang Z., Ohto U., Shibata T., Taoka M., Yamauchi Y., Sato R., Shukla N.M., David S.A., Isobe T., Miyake K. (2018). Structural Analyses of Toll-like Receptor 7 Reveal Detailed RNA Sequence Specificity and Recognition Mechanism of Agonistic Ligands. Cell Rep..

[55] Tanji H., Ohto U., Shibata T., Taoka M., Yamauchi Y., Isobe T., Miyake K., Shimizu T. (2015). Toll-like receptor 8 senses degradation products of single-stranded RNA. Nat. Struct. Mol. Biol..

[56] Wang J.P., Bowen G.N., Padden C., Cerny A., Finberg R.W., Newburger P.E., Kurt-Jones E.A. (2008). Toll-like receptor-mediated activation of neutrophils by influenza A virus. Blood.

[57] De Marcken M., Dhaliwal K., Danielsen A.C., Gautron A.S., Dominguez-Villar M. (2019). TLR7 and TLR8 activate distinct pathways in monocytes during RNA virus infection. Sci. Signal..

[58] Koupenova M., Corkrey H.A., Vitseva O., Manni G., Pang C.J., Clancy L., Yao C., Rade J., Levy D., Wang J.P. (2019). The role of platelets in mediating a response to human influenza infection. Nat. Commun..

[59] Park B.S., Lee J.O. (2013). Recognition of lipopolysaccharide pattern by TLR4 complexes. Exp. Mol. Med..

[60] Shirey K.A., Lai W., Patel M.C., Pletneva L.M., Pang C., Kurt-Jones E., Lipsky M., Roger T., Calandra T., Tracey K.J. (2016). Novel strategies for targeting innate immune responses to influenza. Mucosal Immunol..

[61] Imai Y., Kuba K., Neely G.G., Yaghubian-Malhami R., Perkmann T., van Loo G., Ermolaeva M., Veldhuizen R., Leung Y.H., Wang H. (2008). Identification of oxidative stress and Toll-like receptor 4 signaling as a key pathway of acute lung injury. Cell.

[62] Tsai S.Y., Segovia J.A., Chang T.H., Morris I.R., Berton M.T., Tessier P.A., Tardif M.R., Cesaro A., Bose S. (2014). DAMP molecule S100A9 acts as a molecular pattern to enhance inflammation during influenza A virus infection: Role of DDX21-TRIF-TLR4-MyD88 pathway. PLoS Pathog..

[63] Shirey K.A., Lai W., Scott A.J., Lipsky M., Mistry P., Pletneva L.M., Karp C.L., McAlees J., Gioannini T.L., Weiss J. (2013). The TLR4 antagonist Eritoran protects mice from lethal influenza infection. Nature.

[64] Lee S.M., Kok K.H., Jaume M., Cheung T.K., Yip T.F., Lai J.C., Guan Y., Webster R.G., Jin D.Y., Peiris J.S. (2014). Toll-like receptor 10 is involved in induction of innate immune responses to influenza virus infection. Proc. Natl. Acad. Sci. USA.

[65] Nguyen D.N., Mahon K.P., Chikh G., Kim P., Chung H., Vicari A.P., Love K.T., Goldberg M., Chen S., Krieg A.M. (2012). Lipid-derived nanoparticles for immunostimulatory RNA adjuvant delivery. Proc. Natl. Acad. Sci. USA.

[66] Liu G., Lu Y., Thulasi Raman S.N., Xu F., Wu Q., Li Z., Brownlie R., Liu Q., Zhou Y. (2018). Nuclear-resident RIG-I senses viral replication inducing antiviral immunity. Nat. Commun..

[67] Liu G., Zhou Y. (2019). Cytoplasm and Beyond: Dynamic Innate Immune Sensing of Influenza A Virus by RIG-I. J. Virol..

[68] Schlee M., Roth A., Hornung V., Hagmann C.A., Wimmenauer V., Barchet W., Coch C., Janke M., Mihailovic A., Wardle G. (2009). Recognition of 5′ triphosphate by RIG-I helicase requires short blunt double-stranded RNA as contained in panhandle of negative-strand virus. Immunity.

[69] Schmidt A., Schwerd T., Hamm W., Hellmuth J.C., Cui S., Wenzel M., Hoffmann F.S., Michallet M.-C., Besch R., Hopfner K.-P. (2009). 5′-triphosphate RNA requires base-paired structures to activate antiviral signaling via RIG-I. Proc. Natl. Acad. Sci. USA.

[70] Luo D., Ding S.C., Vela A., Kohlway A., Lindenbach B.D., Pyle A.M. (2011). Structural insights into RNA recognition by RIG-I. Cell.

[71] Brisse M., Ly H. (2019). Comparative Structure and Function Analysis of the RIG-I-Like Receptors: RIG-I and MDA5. Front. Immunol..

[72] Hornung V., Ellegast J., Kim S., Brzózka K., Jung A., Kato H., Poeck H., Akira S., Conzelmann K.-K., Schlee M. (2006). 5′-Triphosphate RNA Is the Ligand for RIG-I. Science.

[73] Goubau D., Schlee M., Deddouche S., Pruijssers A.J., Zillinger T., Goldeck M., Schuberth C., Van der Veen A.G., Fujimura T., Rehwinkel J. (2014). Antiviral immunity via RIG-I-mediated recognition of RNA bearing 5′-diphosphates. Nature.

[74] Schuberth-Wagner C., Ludwig J., Bruder A.K., Herzner A.-M., Zillinger T., Goldeck M., Schmidt T., Schmid-Burgk J.L., Kerber R., Wolter S. (2015). A conserved histidine in the RNA sensor RIG-I controls immune tolerance to N1-2′ O-methylated self RNA. Immunity.

[75] Cui S., Eisenächer K., Kirchhofer A., Brzózka K., Lammens A., Lammens K., Fujita T., Conzelmann K.-K., Krug A., Hopfner K.-P. (2008). The C-terminal regulatory domain is the RNA 5′-triphosphate sensor of RIG-I. Mol. Cell.

[76] Lu C., Xu H., Ranjith-Kumar C., Brooks M.T., Hou T.Y., Hu F., Herr A.B., Strong R.K., Kao C.C., Li P. (2010). The structural basis of 5′ triphosphate double-stranded RNA recognition by RIG-I C-terminal domain. Structure.

[77] Zheng J., Wang C., Chang M.R., Devarkar S.C., Schweibenz B., Crynen G.C., Garcia-Ordonez R.D., Pascal B.D., Novick S.J., Patel S.S. (2018). HDX-MS reveals dysregulated checkpoints that compromise discrimination against self RNA during RIG-I mediated autoimmunity. Nat. Commun..

[78] Kowalinski E., Lunardi T., McCarthy A.A., Louber J., Brunel J., Grigorov B., Gerlier D., Cusack S. (2011). Structural basis for the activation of innate immune pattern-recognition receptor RIG-I by viral RNA. Cell.

[79] Jiang F., Ramanathan A., Miller M.T., Tang G.-Q., Gale M., Patel S.S., Marcotrigiano J. (2011). Structural basis of RNA recognition and activation by innate immune receptor RIG-I. Nature.

[80] Gack M.U., Shin Y.C., Joo C.-H., Urano T., Liang C., Sun L., Takeuchi O., Akira S., Chen Z., Inoue S. (2007). TRIM25 RING-finger E3 ubiquitin ligase is essential for RIG-I-mediated antiviral activity. Nature.

[81] Oshiumi H., Miyashita M., Inoue N., Okabe M., Matsumoto M., Seya T. (2010). The ubiquitin ligase Riplet is essential for RIG-I-dependent innate immune responses to RNA virus infection. Cell Host Microbe.

[82] Davis M.E., Gack M.U. (2015). Ubiquitination in the antiviral immune response. Virology.

[83] Xian H., Xie W., Yang S., Liu Q., Xia X., Jin S., Sun T., Cui J. (2017). Stratified ubiquitination of RIG-I creates robust immune response and induces selective gene expression. Sci. Adv..

[84] Oshiumi H., Miyashita M., Matsumoto M., Seya T. (2013). A distinct role of Riplet-mediated K63-Linked polyubiquitination of the RIG-I repressor domain in human antiviral innate immune responses. PLoS Pathog..

[85] Hayman T.J., Hsu A.C., Kolesnik T.B., Dagley L.F., Willemsen J., Tate M.D., Baker P.J., Kershaw N.J., Kedzierski L., Webb A.I. (2019). RIPLET, and not TRIM25, is required for endogenous RIG-I-dependent antiviral responses. Immunol. Cell Biol..

[86] Wu B., Hur S. (2015). How RIG-I like receptors activate MAVS. Curr Opin. Virol..

[87] Rehwinkel J., Gack M.U. (2020). RIG-I-like receptors: Their regulation and roles in RNA sensing. Nat. Rev. Immunol..

[88] Liu H.M., Loo Y.M., Horner S.M., Zornetzer G.A., Katze M.G., Gale M. (2012). The mitochondrial targeting chaperone 14-3-3ε regulates a RIG-I translocon that mediates membrane association and innate antiviral immunity. Cell Host Microbe.

[89] Chow K.T., Gale M., Loo Y.M. (2018). RIG-I and Other RNA Sensors in Antiviral Immunity. Annu. Rev. Immunol..

[90] Odendall C., Dixit E., Stavru F., Bierne H., Franz K.M., Durbin A.F., Boulant S., Gehrke L., Cossart P., Kagan J.C. (2014). Diverse intracellular pathogens activate type III interferon expression from peroxisomes. Nat. Immunol..

[91] Dixit E., Boulant S., Zhang Y., Lee A.S., Odendall C., Shum B., Hacohen N., Chen Z.J., Whelan S.P., Fransen M. (2010). Peroxisomes are signaling platforms for antiviral innate immunity. Cell.

[92] Michallet M.-C., Meylan E., Ermolaeva M.A., Vazquez J., Rebsamen M., Curran J., Poeck H., Bscheider M., Hartmann G., König M. (2008). TRADD Protein Is an Essential Component of the RIG-like Helicase Antiviral Pathway. Immunity.

[93] Guo B., Cheng G. (2007). Modulation of the interferon antiviral response by the TBK1/IKKi adaptor protein TANK. J. Biol. Chem..

[94] Shi J., Zhao Y., Wang K., Shi X., Wang Y., Huang H., Zhuang Y., Cai T., Wang F., Shao F. (2015). Cleavage of GSDMD by inflammatory caspases determines pyroptotic cell death. Nature.

[95] Rehwinkel J., Tan C.P., Goubau D., Schulz O., Pichlmair A., Bier K., Robb N., Vreede F., Barclay W., Fodor E. (2010). RIG-I detects viral genomic RNA during negative-strand RNA virus infection. Cell.

[96] Liu G., Park H.-S., Pyo H.-M., Liu Q., Zhou Y. (2015). Influenza A Virus Panhandle Structure Is Directly Involved in RIG-I Activation and Interferon Induction. J. Virol..

[97] Baum A., Sachidanandam R., García-Sastre A. (2010). Preference of RIG-I for short viral RNA molecules in infected cells revealed by next-generation sequencing. Proc. Natl. Acad. Sci. USA.

[98] Liu G., Lu Y., Liu Q., Zhou Y. (2019). Inhibition of Ongoing Influenza A Virus Replication Reveals Different Mechanisms of RIG-I Activation. J. Virol..

[99] Te Velthuis A.J.W., Long J.C., Bauer D.L.V., Fan R.L.Y., Yen H.-L., Sharps J., Siegers J.Y., Killip M.J., French H., Oliva-Martín M.J. (2018). Mini viral RNAs act as innate immune agonists during influenza virus infection. Nat. Microbiol..

[100] Perez J.T., Varble A., Sachidanandam R., Zlatev I., Manoharan M., García-Sastre A., tenOever B.R. (2010). Influenza A virus-generated small RNAs regulate the switch from transcription to replication. Proc. Natl. Acad. Sci. USA.

[101] Perez J.T., Zlatev I., Aggarwal S., Subramanian S., Sachidanandam R., Kim B., Manoharan M., tenOever B.R. (2012). A Small-RNA Enhancer of Viral Polymerase Activity. J. Virol..

[102] Kandasamy M., Suryawanshi A., Tundup S., Perez J.T., Schmolke M., Manicassamy S., Manicassamy B. (2016). RIG-I Signaling Is Critical for Efficient Polyfunctional T Cell Responses during Influenza Virus Infection. PLoS Pathog..

[103] Lin L., Liu Q., Berube N., Detmer S., Zhou Y. (2012). 5′-Triphosphate-Short Interfering RNA: Potent Inhibition of Influenza A Virus Infection by Gene Silencing and RIG-I Activation. J. Virol..

[104] Coch C., Stümpel J.P., Lilien-Waldau V., Wohlleber D., Kümmerer B.M., Bekeredjian-Ding I., Kochs G., Garbi N., Herberhold S., Schuberth-Wagner C. (2017). RIG-I Activation Protects and Rescues from Lethal Influenza Virus Infection and Bacterial Superinfection. Mol. Ther..

[105] Newton K., Wickliffe K.E., Maltzman A., Dugger D.L., Strasser A., Pham V.C., Lill J.R., Roose-Girma M., Warming S., Solon M. (2016). RIPK1 inhibits ZBP1-driven necroptosis during development. Nature.

[106] Fu Y., Comella N., Tognazzi K., Brown L.F., Dvorak H.F., Kocher O. (1999). Cloning of DLM-1, a novel gene that is up-regulated in activated macrophages, using RNA differential display. Gene.

[107] Herbert A., Rich A. (1996). The biology of left-handed Z-DNA. J. Biol. Chem..

[108] Ha S.C., Van Quyen D., Hwang H.-Y., Oh D.-B., Brown B.A., Lee S.M., Park H.-J., Ahn J.-H., Kim K.K., Kim Y.-G. (2006). Biochemical characterization and preliminary X-ray crystallographic study of the domains of human ZBP1 bound to left-handed Z-DNA. Biochim. Et Biophys. Acta (BBA)-Proteins Proteom..

[109] Ha S.C., Kim D., Hwang H.-Y., Rich A., Kim Y.-G., Kim K.K. (2008). The crystal structure of the second Z-DNA binding domain of human DAI (ZBP1) in complex with Z-DNA reveals an unusual binding mode to Z-DNA. Proc. Natl. Acad. Sci. USA.

[110] Takaoka A., Wang Z., Choi M.K., Yanai H., Negishi H., Ban T., Lu Y., Miyagishi M., Kodama T., Honda K. (2007). DAI (DLM-1/ZBP1) is a cytosolic DNA sensor and an activator of innate immune response. Nature.

[111] Kaiser W.J., Upton J.W., Mocarski E.S. (2008). Receptor-interacting protein homotypic interaction motif-dependent control of NF-kappa B activation via the DNA-dependent activator of IFN regulatory factors. J. Immunol..

[112] Rebsamen M., Heinz L.X., Meylan E., Michallet M.C., Schroder K., Hofmann K., Vazquez J., Benedict C.A., Tschopp J. (2009). DAI/ZBP1 recruits RIP1 and RIP3 through RIP homotypic interaction motifs to activate NF-κB. EMBO Rep..

[113] Schwartz T., Behlke J., Lowenhaupt K., Heinemann U., Rich A. (2001). Structure of the DLM-1–Z-DNA complex reveals a conserved family of Z-DNA-binding proteins. Nat. Struct. Biol..

[114] Maelfait J., Liverpool L., Bridgeman A., Ragan K.B., Upton J.W., Rehwinkel J. (2017). Sensing of viral and endogenous RNA by ZBP1/DAI induces necroptosis. EMBO J..

[115] Thapa R.J., Ingram J.P., Ragan K.B., Nogusa S., Boyd D.F., Benitez A.A., Sridharan H., Kosoff R., Shubina M., Landsteiner V.J. (2016). DAI Senses Influenza A Virus Genomic RNA and Activates RIPK3-Dependent Cell Death. Cell Host Microbe.

[116] Placido D., Brown B.A., Lowenhaupt K., Rich A., Athanasiadis A. (2007). A left-handed RNA double helix bound by the Zα domain of the RNA-editing enzyme ADAR1. Structure.

[117] Zhang T., Yin C., Boyd D.F., Quarato G., Ingram J.P., Shubina M., Ragan K.B., Ishizuka T., Crawford J.C., Tummers B. (2020). Influenza Virus Z-RNAs Induce ZBP1-Mediated Necroptosis. Cell.

[118] Kuriakose T., Man S.M., Malireddi R.K., Karki R., Kesavardhana S., Place D.E., Neale G., Vogel P., Kanneganti T.D. (2016). ZBP1/DAI is an innate sensor of influenza virus triggering the NLRP3 inflammasome and programmed cell death pathways. Sci. Immunol..

[119] Deigendesch N., Koch-Nolte F., Rothenburg S. (2006). ZBP1 subcellular localization and association with stress granules is controlled by its Z-DNA binding domains. Nucleic Acids Res..

[120] Pham H.T., Park M.-Y., Kim K.K., Kim Y.-G., Ahn J.-H. (2006). Intracellular localization of human ZBP1: Differential regulation by the Z-DNA binding domain, Zα, in splice variants. Biochem. Biophys. Res. Commun..

[121] Rothenburg S., Schwartz T., Koch-Nolte F., Haag F. (2002). Complex regulation of the human gene for the Z-DNA binding protein DLM-1. Nucleic Acids Res..

[122] Jorgensen I., Rayamajhi M., Miao E.A. (2017). Programmed cell death as a defence against infection. Nat. Rev. Immunol..

[123] Pasparakis M., Vandenabeele P. (2015). Necroptosis and its role in inflammation. Nature.

[124] Nogusa S., Thapa R.J., Dillon C.P., Liedmann S., Oguin T.H., Ingram J.P., Rodriguez D.A., Kosoff R., Sharma S., Sturm O. (2016). RIPK3 Activates Parallel Pathways of MLKL-Driven Necroptosis and FADD-Mediated Apoptosis to Protect against Influenza A Virus. Cell Host Microbe.

[125] Moriwaki K., Bertin J., Gough P.J., Chan F.K. (2015). A RIPK3-caspase 8 complex mediates atypical pro-IL-1β processing. J. Immunol..

[126] Walle L.V., Lamkanfi M. (2016). Pyroptosis. Curr. Biol..

[127] Lamkanfi M., Dixit V.M. (2014). Mechanisms and functions of inflammasomes. Cell.

[128] Kesavardhana S., Kuriakose T., Guy C.S., Samir P., Malireddi R.K.S., Mishra A., Kanneganti T.D. (2017). ZBP1/DAI ubiquitination and sensing of influenza vRNPs activate programmed cell death. J. Exp. Med..

[129] Kanneganti T.-D., Lamkanfi M., Núñez G. (2007). Intracellular NOD-like receptors in host defense and disease. Immunity.

[130] Ting J.P.-Y., Lovering R.C., Alnemri E.S., Bertin J., Boss J.M., Davis B.K., Flavell R.A., Girardin S.E., Godzik A., Harton J.A. (2008). The NLR gene family: A standard nomenclature. Immunity.

[131] Lechtenberg B.C., Mace P.D., Riedl S.J. (2014). Structural mechanisms in NLR inflammasome signaling. Curr. Opin. Struct. Biol..

[132] Swanson K.V., Deng M., Ting J.P. (2019). The NLRP3 inflammasome: Molecular activation and regulation to therapeutics. Nat. Rev. Immunol..

[133] Bauernfeind F.G., Horvath G., Stutz A., Alnemri E.S., MacDonald K., Speert D., Fernandes-Alnemri T., Wu J., Monks B.G., Fitzgerald K.A. (2009). Cutting edge: NF-kappaB activating pattern recognition and cytokine receptors license NLRP3 inflammasome activation by regulating NLRP3 expression. J. Immunol..

[134] Franchi L., Eigenbrod T., Núñez G. (2009). Cutting edge: TNF-alpha mediates sensitization to ATP and silica via the NLRP3 inflammasome in the absence of microbial stimulation. J. Immunol..

[135] Xing Y., Yao X., Li H., Xue G., Guo Q., Yang G., An L., Zhang Y., Meng G. (2017). Cutting edge: TRAF6 mediates TLR/IL-1R signaling–induced nontranscriptional priming of the NLRP3 inflammasome. J. Immunol..

[136] Dick M.S., Sborgi L., Rühl S., Hiller S., Broz P. (2016). ASC filament formation serves as a signal amplification mechanism for inflammasomes. Nat. Commun..

[137] Cai X., Chen J., Xu H., Liu S., Jiang Q.-X., Halfmann R., Chen Z.J. (2014). Prion-like polymerization underlies signal transduction in antiviral immune defense and inflammasome activation. Cell.

[138] Lu A., Magupalli V.G., Ruan J., Yin Q., Atianand M.K., Vos M.R., Schröder G.F., Fitzgerald K.A., Wu H., Egelman E.H. (2014). Unified polymerization mechanism for the assembly of ASC-dependent inflammasomes. Cell.

[139] Boucher D., Monteleone M., Coll R.C., Chen K.W., Ross C.M., Teo J.L., Gomez G.A., Holley C.L., Bierschenk D., Stacey K.J. (2018). Caspase-1 self-cleavage is an intrinsic mechanism to terminate inflammasome activity. J. Exp. Med..

[140] Broz P., Dixit V. (2016). Inflammasomes: Mechanism of assembly, regulation and signalling. Nat. Rev. Immunol..

[141] Rathinam V.A., Fitzgerald K.A. (2016). Inflammasome Complexes: Emerging Mechanisms and Effector Functions. Cell.

[142] Niu J., Wu S., Chen M., Xu K., Guo Q., Lu A., Zhao L., Sun B., Meng G. (2019). Hyperactivation of the NLRP3 inflammasome protects mice against influenza A virus infection via IL-1β mediated neutrophil recruitment. Cytokine.

[143] Joosten L.A., Netea M.G., Dinarello C.A. (2013). Interleukin-1β in innate inflammation, autophagy and immunity. Semin Immunol..

[144] Dinarello C.A., Novick D., Kim S., Kaplanski G. (2013). Interleukin-18 and IL-18 binding protein. Front. Immunol..

[145] Schmid-Burgk J.L., Chauhan D., Schmidt T., Ebert T.S., Reinhardt J., Endl E., Hornung V. (2015). A genome-wide CRISPR screen identifies NEK7 as an essential component of NLRP3 inflammasome activation. J. Biol. Chem..

[146] He Y., Zeng M.Y., Yang D., Motro B., Núñez G. (2016). NEK7 is an essential mediator of NLRP3 activation downstream of potassium efflux. Nature.

[147] Muñoz-Planillo R., Kuffa P., Martínez-Colón G., Smith B.L., Rajendiran T.M., Núñez G. (2013). K⁺ efflux is the common trigger of NLRP3 inflammasome activation by bacterial toxins and particulate matter. Immunity.

[148] Pétrilli V., Papin S., Dostert C., Mayor A., Martinon F., Tschopp J. (2007). Activation of the NALP3 inflammasome is triggered by low intracellular potassium concentration. Cell Death Differ..

[149] Tang T., Lang X., Xu C., Wang X., Gong T., Yang Y., Cui J., Bai L., Wang J., Jiang W. (2017). CLICs-dependent chloride efflux is an essential and proximal upstream event for NLRP3 inflammasome activation. Nat. Commun..

[150] Murakami T., Ockinger J., Yu J., Byles V., McColl A., Hofer A.M., Horng T. (2012). Critical role for calcium mobilization in activation of the NLRP3 inflammasome. Proc. Natl. Acad. Sci. USA.

[151] Lee G.S., Subramanian N., Kim A.I., Aksentijevich I., Goldbach-Mansky R., Sacks D.B., Germain R.N., Kastner D.L., Chae J.J. (2012). The calcium-sensing receptor regulates the NLRP3 inflammasome through Ca2+ and cAMP. Nature.

[152] Zhang Q., Raoof M., Chen Y., Sumi Y., Sursal T., Junger W., Brohi K., Itagaki K., Hauser C.J. (2010). Circulating mitochondrial DAMPs cause inflammatory responses to injury. Nature.

[153] Zhong Z., Liang S., Sanchez-Lopez E., He F., Shalapour S., Lin X.-J., Wong J., Ding S., Seki E., Schnabl B. (2018). New mitochondrial DNA synthesis enables NLRP3 inflammasome activation. Nature.

[154] Shimada K., Crother T.R., Karlin J., Dagvadorj J., Chiba N., Chen S., Ramanujan V.K., Wolf A.J., Vergnes L., Ojcius D.M. (2012). Oxidized mitochondrial DNA activates the NLRP3 inflammasome during apoptosis. Immunity.

[155] Zhou R., Yazdi A.S., Menu P., Tschopp J. (2011). A role for mitochondria in NLRP3 inflammasome activation. Nature.

[156] Rawat P., Teodorof-Diedrich C., Spector S.A. (2019). Human immunodeficiency virus Type-1 single-stranded RNA activates the NLRP3 inflammasome and impairs autophagic clearance of damaged mitochondria in human microglia. Glia.

[157] Chen J., Chen Z.J. (2018). PtdIns4P on dispersed trans-Golgi network mediates NLRP3 inflammasome activation. Nature.

[158] Mitoma H., Hanabuchi S., Kim T., Bao M., Zhang Z., Sugimoto N., Liu Y.-J. (2013). The DHX33 RNA helicase senses cytosolic RNA and activates the NLRP3 inflammasome. Immunity.

[159] Li J., Hu L., Liu Y., Huang L., Mu Y., Cai X., Weng C. (2015). DDX19A Senses Viral RNA and Mediates NLRP3-Dependent Inflammasome Activation. J. Immunol..

[160] Pothlichet J., Meunier I., Davis B.K., Ting J.P., Skamene E., von Messling V., Vidal S.M. (2013). Type I IFN triggers RIG-I/TLR3/NLRP3-dependent inflammasome activation in influenza A virus infected cells. PLoS Pathog..

[161] Thomas P.G., Dash P., Aldridge J.R., Ellebedy A.H., Reynolds C., Funk A.J., Martin W.J., Lamkanfi M., Webby R.J., Boyd K.L. (2009). The Intracellular Sensor NLRP3 Mediates Key Innate and Healing Responses to Influenza A Virus via the Regulation of Caspase-1. Immunity.

[162] Allen I.C., Scull M.A., Moore C.B., Holl E.K., McElvania-TeKippe E., Taxman D.J., Guthrie E.H., Pickles R.J., Ting J.P. (2009). The NLRP3 inflammasome mediates in vivo innate immunity to influenza A virus through recognition of viral RNA. Immunity.

[163] Ichinohe T., Pang I.K., Iwasaki A. (2010). Influenza virus activates inflammasomes via its intracellular M2 ion channel. Nat. Immunol..

[164] Ju X., Yan Y., Liu Q., Li N., Sheng M., Zhang L., Li X., Liang Z., Huang F., Liu K. (2015). Neuraminidase of Influenza A Virus Binds Lysosome-Associated Membrane Proteins Directly and Induces Lysosome Rupture. J. Virol..

[165] McGuire K.A., Barlan A.U., Griffin T.M., Wiethoff C.M. (2011). Adenovirus type 5 rupture of lysosomes leads to cathepsin B-dependent mitochondrial stress and production of reactive oxygen species. J. Virol..

[166] McAuley J.L., Tate M.D., MacKenzie-Kludas C.J., Pinar A., Zeng W., Stutz A., Latz E., Brown L.E., Mansell A. (2013). Activation of the NLRP3 inflammasome by IAV virulence protein PB1-F2 contributes to severe pathophysiology and disease. PLoS Pathog..

[167] Yoshizumi T., Ichinohe T., Sasaki O., Otera H., Kawabata S., Mihara K., Koshiba T. (2014). Influenza A virus protein PB1-F2 translocates into mitochondria via Tom40 channels and impairs innate immunity. Nat. Commun..

[168] Park H.S., Liu G., Liu Q., Zhou Y. (2018). Swine Influenza Virus Induces RIPK1/DRP1-Mediated Interleukin-1 Beta Production. Viruses.

[169] Chakrabarti A., Banerjee S., Franchi L., Loo Y.M., Gale M., Núñez G., Silverman R.H. (2015). RNase L activates the NLRP3 inflammasome during viral infections. Cell Host Microbe.

[170] Ichinohe T., Pang I.K., Kumamoto Y., Peaper D.R., Ho J.H., Murray T.S., Iwasaki A. (2011). Microbiota regulates immune defense against respiratory tract influenza A virus infection. Proc. Natl. Acad. Sci. USA.

[171] Sabbah A., Chang T.H., Harnack R., Frohlich V., Tominaga K., Dube P.H., Xiang Y., Bose S. (2009). Activation of innate immune antiviral responses by Nod2. Nat. Immunol..

[172] Hale B.G., Randall R.E., Ortín J., Jackson D. (2008). The multifunctional NS1 protein of influenza A viruses. J. Gen. Virol..

[173] Marc D. (2014). Influenza virus non-structural protein NS1: Interferon antagonism and beyond. J. Gen. Virol..

[174] García-Sastre A., Egorov A., Matassov D., Brandt S., Levy D.E., Durbin J.E., Palese P., Muster T. (1998). Influenza A Virus Lacking the NS1 Gene Replicates in Interferon-Deficient Systems. Virology.

[175] Jureka A.S., Kleinpeter A.B., Tipper J.L., Harrod K.S., Petit C.M. (2020). The influenza NS1 protein modulates RIG-I activation via a strain-specific direct interaction with the second CARD of RIG-I. J. Biol. Chem..

[176] Mibayashi M., Martínez-Sobrido L., Loo Y.-M., Cárdenas W.B., Gale M., García-Sastre A. (2007). Inhibition of Retinoic Acid-Inducible Gene I-Mediated Induction of Beta Interferon by the NS1 Protein of Influenza A Virus. J. Virol..

[177] Guo Z., Chen L.M., Zeng H., Gomez J.A., Plowden J., Fujita T., Katz J.M., Donis R.O., Sambhara S. (2007). NS1 protein of influenza A virus inhibits the function of intracytoplasmic pathogen sensor, RIG-I. Am. J. Respir. Cell Mol. Biol..

[178] Gack M.U., Albrecht R.A., Urano T., Inn K.S., Huang I.C., Carnero E., Farzan M., Inoue S., Jung J.U., García-Sastre A. (2009). Influenza A virus NS1 targets the ubiquitin ligase TRIM25 to evade recognition by the host viral RNA sensor RIG-I. Cell Host Microbe.

[179] Rajsbaum R., Albrecht R.A., Wang M.K., Maharaj N.P., Versteeg G.A., Nistal-Villán E., García-Sastre A., Gack M.U. (2012). Species-Specific Inhibition of RIG-I Ubiquitination and IFN Induction by the Influenza A Virus NS1 Protein. PLoS Pathog..

[180] Koliopoulos M.G., Lethier M., van der Veen A.G., Haubrich K., Hennig J., Kowalinski E., Stevens R.V., Martin S.R., Reis e Sousa C., Cusack S. (2018). Molecular mechanism of influenza A NS1-mediated TRIM25 recognition and inhibition. Nat. Commun..

[181] Feng W., Sun X., Shi N., Zhang M., Guan Z., Duan M. (2017). Influenza a virus NS1 protein induced A20 contributes to viral replication by suppressing interferon-induced antiviral response. Biochem. Biophys. Res. Commun..

[182] Maelfait J., Roose K., Vereecke L., Mc Guire C., Sze M., Schuijs M.J., Willart M., Itati Ibañez L., Hammad H., Lambrecht B.N. (2016). A20 Deficiency in Lung Epithelial Cells Protects against Influenza A Virus Infection. PLoS Pathog..

[183] Park H.-S., Liu G., Thulasi Raman S.N., Landreth S.L., Liu Q., Zhou Y. (2018). NS1 Protein of 2009 Pandemic Influenza A Virus Inhibits Porcine NLRP3 Inflammasome-Mediated Interleukin-1 Beta Production by Suppressing ASC Ubiquitination. J. Virol..

[184] Moriyama M., Chen I.-Y., Kawaguchi A., Koshiba T., Nagata K., Takeyama H., Hasegawa H., Ichinohe T. (2016). The RNA- and TRIM25-Binding Domains of Influenza Virus NS1 Protein Are Essential for Suppression of NLRP3 Inflammasome-Mediated Interleukin-1β Secretion. J. Virol..

[185] Gaba A., Xu F., Lu Y., Park H.-S., Liu G., Zhou Y., López S. (2019). The NS1 Protein of Influenza A Virus Participates in Necroptosis by Interacting with MLKL and Increasing Its Oligomerization and Membrane Translocation. J. Virol..

[186] Gao S., Song L., Li J., Zhang Z., Peng H., Jiang W., Wang Q., Kang T., Chen S., Huang W. (2012). Influenza A virus-encoded NS1 virulence factor protein inhibits innate immune response by targeting IKK. Cell. Microbiol..

[187] Wang X., Li M., Zheng H., Muster T., Palese P., Beg A.A., García-Sastre A. (2000). Influenza A Virus NS1 Protein Prevents Activation of NF-κB and Induction of Alpha/Beta Interferon. J. Virol..

[188] Ludwig S., Wang X., Ehrhardt C., Zheng H., Donelan N., Planz O., Pleschka S., García-Sastre A., Heins G., Wolff T. (2002). The Influenza A Virus NS1 Protein Inhibits Activation of Jun N-Terminal Kinase and AP-1 Transcription Factors. J. Virol..

[189] Nemeroff M.E., Barabino S.M., Li Y., Keller W., Krug R.M. (1998). Influenza virus NS1 protein interacts with the cellular 30 kDa subunit of CPSF and inhibits 3′end formation of cellular pre-mRNAs. Mol. Cell.

[190] Fortes P., Beloso A., Ortín J. (1994). Influenza virus NS1 protein inhibits pre-mRNA splicing and blocks mRNA nucleocytoplasmic transport. EMBO J..

[191] Qiu Y., Nemeroff M., Krug R.M. (1995). The influenza virus NS1 protein binds to a specific region in human U6 snRNA and inhibits U6-U2 and U6-U4 snRNA interactions during splicing. RNA.

[192] Satterly N., Tsai P.L., van Deursen J., Nussenzveig D.R., Wang Y., Faria P.A., Levay A., Levy D.E., Fontoura B.M. (2007). Influenza virus targets the mRNA export machinery and the nuclear pore complex. Proc. Natl. Acad. Sci. USA.

[193] Chen W., Calvo P.A., Malide D., Gibbs J., Schubert U., Bacik I., Basta S., O’Neill R., Schickli J., Palese P. (2001). A novel influenza A virus mitochondrial protein that induces cell death. Nat. Med..

[194] Chakrabarti A.K., Pasricha G. (2013). An insight into the PB1F2 protein and its multifunctional role in enhancing the pathogenicity of the influenza A viruses. Virology.

[195] Zell R., Krumbholz A., Eitner A., Krieg R., Halbhuber K.J., Wutzler P. (2007). Prevalence of PB1-F2 of influenza A viruses. J. Gen. Virol..

[196] Kamal R.P., Kumar A., Davis C.T., Tzeng W.P., Nguyen T., Donis R.O., Katz J.M., York I.A. (2015). Emergence of Highly Pathogenic Avian Influenza A(H5N1) Virus PB1-F2 Variants and Their Virulence in BALB/c Mice. J. Virol..

[197] Koshiba T., Yasukawa K., Yanagi Y., Kawabata S. (2011). Mitochondrial membrane potential is required for MAVS-mediated antiviral signaling. Sci. Signal..

[198] Ichinohe T., Yamazaki T., Koshiba T., Yanagi Y. (2013). Mitochondrial protein mitofusin 2 is required for NLRP3 inflammasome activation after RNA virus infection. Proc. Natl. Acad. Sci. USA.

[199] Varga Z.T., Ramos I., Hai R., Schmolke M., García-Sastre A., Fernandez-Sesma A., Palese P. (2011). The influenza virus protein PB1-F2 inhibits the induction of type I interferon at the level of the MAVS adaptor protein. PLoS Pathog..

[200] Varga Z.T., Grant A., Manicassamy B., Palese P. (2012). Influenza virus protein PB1-F2 inhibits the induction of type I interferon by binding to MAVS and decreasing mitochondrial membrane potential. J. Virol..

[201] Dudek S.E., Wixler L., Nordhoff C., Nordmann A., Anhlan D., Wixler V., Ludwig S. (2011). The influenza virus PB1-F2 protein has interferon antagonistic activity. Biol. Chem..

[202] Gloire G., Horion J., El Mjiyad N., Bex F., Chariot A., Dejardin E., Piette J. (2007). Promoter-dependent effect of IKKα on NF-κB/p65 DNA binding. J. Biol. Chem..

[203] Reis A.L., McCauley J.W. (2013). The influenza virus protein PB1-F2 interacts with IKKβ and modulates NF-κB signalling. PLoS ONE.

[204] Leymarie O., Meyer L., Tafforeau L., Lotteau V., Costa B.D., Delmas B., Chevalier C., Le Goffic R. (2017). Influenza virus protein PB1-F2 interacts with CALCOCO2 (NDP52) to modulate innate immune response. J. Gen. Virol..

[205] Thurston T.L.M., Ryzhakov G., Bloor S., von Muhlinen N., Randow F. (2009). The TBK1 adaptor and autophagy receptor NDP52 restricts the proliferation of ubiquitin-coated bacteria. Nat. Immunol..

[206] Firth A., Jagger B., Wise H., Nelson C., Parsawar K., Wills N., Napthine S., Taubenberger J., Digard P., Atkins J. (2012). Ribosomal frameshifting used in influenza A virus expression occurs within the sequence UCC_UUU_CGU and is in the+ 1 direction. Open Biol..

[207] Jagger B.W., Wise H.M., Kash J.C., Walters K.A., Wills N.M., Xiao Y.L., Dunfee R.L., Schwartzman L.M., Ozinsky A., Bell G.L. (2012). An overlapping protein-coding region in influenza A virus segment 3 modulates the host response. Science.

[208] Levene R.E., Gaglia M.M. (2018). Host Shutoff in Influenza A Virus: Many Means to an End. Viruses.

[209] Khaperskyy D.A., Schmaling S., Larkins-Ford J., McCormick C., Gaglia M.M. (2016). Selective Degradation of Host RNA Polymerase II Transcripts by Influenza A Virus PA-X Host Shutoff Protein. PLoS Pathog..

[210] Khaperskyy D.A., McCormick C. (2015). Timing Is Everything: Coordinated Control of Host Shutoff by Influenza A Virus NS1 and PA-X Proteins. J. Virol..

[211] Hayashi T., Chaimayo C., McGuinness J., Takimoto T. (2016). Critical Role of the PA-X C-Terminal Domain of Influenza A Virus in Its Subcellular Localization and Shutoff Activity. J. Virol..

[212] Gaucherand L., Porter B.K., Levene R.E., Price E.L., Schmaling S.K., Rycroft C.H., Kevorkian Y., McCormick C., Khaperskyy D.A., Gaglia M.M. (2019). The Influenza A Virus Endoribonuclease PA-X Usurps Host mRNA Processing Machinery to Limit Host Gene Expression. Cell Rep..

[213] Hayashi T., MacDonald L.A., Takimoto T. (2015). Influenza A Virus Protein PA-X Contributes to Viral Growth and Suppression of the Host Antiviral and Immune Responses. J. Virol..

[214] Rigby R.E., Wise H.M., Smith N., Digard P., Rehwinkel J. (2019). PA-X antagonises MAVS-dependent accumulation of early type I interferon messenger RNAs during influenza A virus infection. Sci. Rep..

[215] Weber M., Sediri H., Felgenhauer U., Binzen I., Bänfer S., Jacob R., Brunotte L., García-Sastre A., Schmid-Burgk J.L., Schmidt T. (2015). Influenza Virus Adaptation PB2-627K Modulates Nucleocapsid Inhibition by the Pathogen Sensor RIG-I. Cell Host Microbe.

[216] Huang S., Zhu B., Cheon I.S., Goplen N.P., Jiang L., Zhang R., Peebles R.S., Mack M., Kaplan M.H., Limper A.H. (2019). PPAR-γ in Macrophages Limits Pulmonary Inflammation and Promotes Host Recovery following Respiratory Viral Infection. J. Virol..

[217] Xia Z., Xu G., Yang X., Peng N., Zuo Q., Zhu S., Hao H., Liu S., Zhu Y. (2017). Inducible TAP1 Negatively Regulates the Antiviral Innate Immune Response by Targeting the TAK1 Complex. J. Immunol..

[218] Allen I.C., Moore C.B., Schneider M., Lei Y., Davis B.K., Scull M.A., Gris D., Roney K.E., Zimmermann A.G., Bowzard J.B. (2011). NLRX1 protein attenuates inflammatory responses to infection by interfering with the RIG-I-MAVS and TRAF6-NF-κB signaling pathways. Immunity.

[219] Si-Tahar M., Blanc F., Furio L., Chopy D., Balloy V., Lafon M., Chignard M., Fiette L., Langa F., Charneau P. (2014). Protective role of LGP2 in influenza virus pathogenesis. J. Infect. Dis..

